# Phyllosphere Fungal Communities of Plum and Antifungal Activity of Indigenous Phenazine-Producing *Pseudomonas synxantha* Against *Monilinia laxa*

**DOI:** 10.3389/fmicb.2019.02287

**Published:** 2019-10-01

**Authors:** Tamara Janakiev, Ivica Dimkić, Nikola Unković, Milica Ljaljević Grbić, Dejan Opsenica, Uroš Gašić, Slaviša Stanković, Tanja Berić

**Affiliations:** ^1^Faculty of Biology, University of Belgrade, Belgrade, Serbia; ^2^Institute of Chemistry, Technology and Metallurgy, University of Belgrade, Belgrade, Serbia; ^3^Faculty of Chemistry, University of Belgrade, Belgrade, Serbia

**Keywords:** NGS, fungal diversity, plum cultivars, biocontrol, *Monilinia laxa*, *Pseudomonas synxantha*

## Abstract

European plum (*Prunus domestica* L.) is a significant commercial crop in Serbia in terms of total fruit production, and is traditionally processed into slivovitz brandy. The brown rot disease caused by *Monilinia laxa* drastically reduces plum yield almost every year. Fungal communities associated with leaves and fruits of four local Serbian plum cultivars (Požegača, Ranka, Čačanska Lepotica and Čačanska Rodna) were investigated in two phenological stages during early (May) and late (July) fruit maturation. Alpha diversity indices showed that fungal communities were heterogeneous and Beta diversity indicated that autochthonous fungal communities depended upon seasonal changes and the cultivars themselves. The phylum Ascomycota was the most abundant in all samples, with relative abundance (RA) between 46% in the Požegača cultivar (May) and 89% in the Lepotica cultivar (July). The most abundant genus for all plum cultivars in May was *Aureobasidium*, with RA from 19.27 to 33.69%, followed by *Cryptococcus*, with 4.8 to 48.80%. In July, besides *Cryptococcus*, different genera (*Metschnikowia*, *Fusarium*, and *Hanseniaspora*) were dominant on particular cultivars. Among all cultivable fungi, molecular identification of eleven *M. laxa* isolates from four plum cultivars was performed simultaneously. Bacterial isolates from the plum phyllosphere were tested for their potential antifungal activity against indigenous *M. laxa* isolates. The most potent antagonist P4/16_1, which significantly reduced mycelial growth of *M. laxa*, was identified as *Pseudomonas synxantha*. Further characterization of P4/16_1 revealed the production of volatile organic compounds and phenazine-1-carboxylic acid (PCA). Crude benzene extract of PCA exhibited 57–63% mycelial growth inhibition of *M. laxa*. LC/MS analysis of the crude extract confirmed the presence of phenazine derivatives amongst other compounds. Scanning electron microscopy revealed morpho-physiological changes in the hyphae of *M. laxa* isolates caused by the cell culture and the *P. synxantha* P4/16_1 crude benzene extract. This is the first report of antagonistic activity of *P. synxantha* against *M. laxa* induced by diffusible and volatile antifungal compounds, and it appears to be a promising candidate for further investigation for potential use as a biocontrol agent against brown rot-causing fungi.

## Introduction

The phyllosphere represents the surface and interior of aerial parts of plants, including flowers, fruits, stems and leaves (Magan, 2006). Besides plant pathogens, which were mainly in the focus of previous investigations, most phyllosphere-colonizing microorganisms live as commensals on their host plants. They can contribute to plant health and development as biofertilizers, phytostimulators, and biopesticides (Newton et al., 2010; Müller and Ruppel, 2014). In recent years, metagenomic analyses have been increasingly utilized to investigate microbial diversity in a number of different environments, but have mainly been focused on soils and the rhizosphere. Fungal biodiversity in the phyllosphere has recently been the focus of several investigations and has been analyzed on tomato, grapevine, balsam poplar, olive, apple, and strawberry (Ottesen et al., 2013; Pinto et al., 2014; Abdelfattah et al., 2015, 2016).

The genus *Prunus* is composed of hundreds of species, including *Prunus domestica*, known as the European plum (Lino et al., 2016). Knowledge of the fungal ecology of plums is often limited to reports indicating the presence of individual microorganisms at a given time. Previous studies investigated fungal communities using conventional cultivation methods, mainly based on isolation of fungi causing brown rot (Poniatowska et al., 2013; Hrustić et al., 2015). There are no reports about the use of the metabarcoding approach for the assessment of fungal diversity in the plum phyllosphere. Plum is a significant commercial crop worldwide (Leff et al., 2004) and in Serbia, where it is most important for total fruit production and for production of slivovitz brandy (Hrustić et al., 2015). Most of the annual production of plum in Serbia (80%) is processed for making this important brandy (Matković, 2015). Brown rot, the most devastating and economically most important disease of stone fruits, is caused by *Monilinia* spp. under favorable warm, wet and humid conditions, causing significant crop losses worldwide. The three main *Monilinia* species causing brown rot are *M. fructicola* (G. Winter) Honey, *M. laxa* (Aderh. & Ruhland) Honey and *M. fructigena* (Aderh. & Ruhland) Honey (Rungjindamai et al., 2014).

Brown rot is controlled by fungicide applications, which poses challenges of sustainability in growing of stone fruit crops, as there are many fungicide-resistant strains (Lino et al., 2016). Also, in recent years there is a globally recognized need to find safe alternatives to pesticide use in agriculture, in view of its negative impact on human health and the environment (Raio et al., 2017). Biological control, which includes use of microorganisms with antagonistic activity against pathogens, is one of the alternative control methods in pest management (Janisiewicz et al., 2000). The epiphyte microflora on plant material is a good source of antagonistic microorganisms. More so, the richest source of antagonists against fruit decay is the fruit microflora, the active ingredient in all currently available commercial biocontrol products (Janisiewicz et al., 2013). One of the first formulated postharvest biocontrol products, now widely used, BioSave^TM^ (JetHarvest Solutions) is based on a saprophytic strain of *Pseudomonas syringae* active against number of postharvest decaying fungi, but not *Monilinia*. Many studies since than reported antagonists effective against *M. laxa*, including *Aureobasidium pullulans* (Mari et al., 2012; Rungjindamai et al., 2013; Di Francesco et al., 2017); *Pantoea agglomerans* (Bonaterra et al., 2003); and *Bacillus amyloliquefaciens* (Rungjindamai et al., 2013) as well as some *Pseudomonas* spp. (Altindag et al., 2006).

Bacteria of the genus *Pseudomonas* are commonly found among the predominant genera in the phyllosphere. The antagonistic activity of fluorescent pseudomonads has been investigated in a few studies of bacteria in the phyllosphere, e.g., in that of apple and pear (Müller et al., 2016). Various studies have reported the ability of members of the genus *Pseudomonas* to control fungal diseases (Santoyo et al., 2012). *Pseudomonas* spp. produce a wide variety of bioactive compounds involved in pathogen control. These include siderophores, hydrogen cyanide, 2,4-diacetylphloroglucinol, pyrrolnitrin, pyoluteorin, phenazines, 2,5-dialkylresorcinol, quinolones, gluconic acid, rhamnolipids and cyclic lipopeptides, as well as excreted hydrolytic enzymes such as proteases, cellulase, chitinase, and β-glucanase (Janisiewicz and Roitman, 1988; Weller, 2007; Gross and Loper, 2009; D’aes et al., 2010). *Pseudomonas synxantha* is a fluorescent bacterium of the *P. fluorescens* group (Wechter et al., 2002). Mukherjee et al. (2014) reported antibacterial activity of bioactive compounds from *P. synxantha* against representatives of the genus *Mycobacterium*. Its antifungal activity was most recently evaluated against *M. fructigena* and *M. fructicola* (Aiello et al., 2019).

The present study gives for the first time an inventory of fungal communities associated with the phyllosphere of four plum cultivars in different phenological phases. In addition, bacterial and yeast isolates from the plum phyllosphere are tested for their potential antifungal activity on indigenous brown rot–causing *Monilinia* isolates *in vitro*. Phenazine-producing *P. synxantha* isolate showed potential to reduce brown rot making it a good candidate for further investigation for possible application in biocontrol.

## Materials and Methods

### Plum Cultivars, Isolation of Fungi and Their Characterization

Samples of leaves and fruits from four local Serbian plum cultivars, the autochthonous varieties Požegača and Ranka and the grafted varieties Čačanska Lepotica (hereafter referred to as Lepotica) and Čačanska Rodna (hereafter referred to as Rodna), were collected from an orchard not treated with pesticides in the last 10 years, located in Saraorci (municipality of Smederevo, Serbia; 44°29′ 11′′ N and 21°04′34′′E) during 2016. Sampling was conducted during different phenological stages of leaves and fruits, in May and July. Leaves and fruits with symptoms of fungal infection were collected aseptically, put in plastic bags and transported to the laboratory in a cooler box. For each cultivar, samples were collected from three trees, and every sample consisted of five leaves and five fruits. Fungal isolation was conducted according to a procedure described previously (Janisiewicz et al., 2013). Plant material was washed with 200 mL of sterile 1 × phosphate-buffered saline (PBS, Sigma Aldrich, United Kingdom) in 1000-mL beakers by shaking on a rotary shaker for 3 min. The washings were discarded, fresh buffer was added and plant material was sonicated for 1 min in a sonication bath and then shaken for 15 min. For fungal isolation, 100 μL of PBS washing was plated on potato dextrose agar (PDA), in addition to which whole leaves were placed on PDA, and incubated for 7 days at 25°C. *Monilinia* isolates were selected specifically from among other fungi based on the following characteristics: color, texture and pigmentation of the colony; the presence of conidia; and the growth pattern after 7 days of incubation at 25°C. Pure cultures of the selected fungal isolates were maintained on PDA slants at 4°C until further use.

### Molecular Identification of *Monilinia* spp.

Molecular identification was conducted for 11 putative Monilinia isolates originating from all cultivars. Prior to genomic DNA extraction, 100 mg mycelia were collected from 7-day old PDA-grown Monilinia cultures and re-suspended in 200 μl of sterile water. Genomic DNA was isolated using the commercial ZR Fungal/Bacterial DNA MiniPrepTM Kit according to the manufacturer’s instructions (Zymo Research, United States). Molecular identification was performed by amplifying two gene regions: ITS1 (partial 18S + internal transcribed spacer 1 and partial 5.8S) and ITS2 (partial 5.8S + internal transcribed spacer 2 + partial 28S). Primer sequences used were as follows: ITS1-F (TCCGTAGGTGAACCTGCGG) and ITS4-R (TCCTCCGCTTATTGATATGC) (White et al., 1990); and ITS3-F (GCATCGATGAAGAACGCAGC) and TW13-R (GGTCCGTGTTTCAAGACG) (White et al., 1990; Tedersoo et al., 2015). PCR reactions were performed as described by Unković et al. (2017) as follows: one denaturation cycle at 94°C for 4 min, followed by 30 cycles of denaturation at 94°C for 30 s, annealing at 55°C for 50 s and extension at 72°C for 50 s, with one final cycle of extension at 72°C for 10 min. PCR products were purified using the QIAquick PCR Purification Kit according to manufacturer’s protocol (Qiagen, Germany) and later sequenced by Macrogen, Inc. (Amsterdam, Netherlands).

All chromatograms were checked manually after which sequences obtained were subjected to homology search on GenBank via BLASTN (BLASTN, RRID:SCR_001598) of the National Centre for Biotechnology Information (NCBI)., Edited and reference strain sequences of *M. laxa, M. fructigena, M. fructicola*, and *M. polystroma* from the GenBank database were aligned using CLUSTAL W (Thompson et al., 1997) implemented in BioEdit 7.2.6 software (BioEdit, RRID:SCR_007361). Phylogenetic trees were constructed in MEGA 6.0 software (MEGA Software, RRID:SCR_000667) using the neighbor-joining method based on a pairwise distance matrix obtained with the Kimura two-parameter nucleotide substitution model. The topology of the trees was evaluated by the bootstrap resampling method with 1000 replicates. The ITS1 and ITS2 sequences of *Sclerotinia sclerotiorum* (GenBank accessions: KM272342 and KC311494) were included as outgroup references.

### Extraction of DNA, Library Preparation and NGS Sequencing

Samples prepared for isolation of culturable fungi were also used for metagenomic analysis. Each sample contained a pool of five leaves and five fruits, collected from three individual trees. Plant material from each sample was washed using1 × PBS. Washings were used for extraction of total DNA from the plum phyllosphere. For each sample, 100 mL of PBS from plant material washing was filtered with Isopore^TM^ membrane filters (Merck Millipore Ltd., Ireland). Total DNA was isolated from four plum cultivars in two phenological stages. The DNA was extracted from 0.22-μm polycarbonate Isopore^TM^ filters using the ZymoBIOMICS^TM^ DNA Mini Kit (Zymo Research, United States) following the manufacturer’s instructions. Subsequently, DNA was quantified by Qubit fluorometric quantitation (a Qubit 4 fluorometer from Invitrogen^TM^, United States) after which DNA samples were dissolved in DNase/RNase-free water and commercially sequenced by Macrogen, Inc. (Seoul, South Korea). The amplicon libraries were amplified using qPCR according to the Illumina qPCR Quantification Protocol Guide (lllumina ITS2 3F-4R amplicon library preparation). The size measured on an Agilent Technologies 2100 bioanalyser using a DNA 1000 chip was between 498 and 574 bp, with an amplicon concentration of between 114 and 135 ng/μL. After size verification, the libraries were sequenced using a 2 × 300-bp paired-end run [MiSeq Reagent Kit, v. 3 (MS-102-3001)] on an MiSeq sequencer according to instructions of the manufacturer (Illumina).

### Processing of NGS Sequence Data, Taxonomy Annotation and Bioinformatic Analysis

Quality assessment and sequence joining were performed using the Prinseq-lite program (Schmieder and Edwards, 2011) with a minimum length of 50, trimming the 3-prime end with a threshold of 30, the mean type of quality score and a trimming quality window of 20. Data analysis was conducted using an *ad hoc* pipeline in Rstatistics (R Development Core Team, 2012). The DADA2 pipeline was used for denoising, paired-end joining and chimera depletion, starting from the paired-ends data (Callahan et al., 2016). Taxonomic affiliations were assigned using the Naive Bayesian classifier integrated in QIIME2 (QIIME, RRID:SCR_008249) plug-ins with taxonomy assignment to the SILVA 132 release (Quast et al., 2013). Sequences ascribed to chloroplasts and to unidentified fungi were excluded from further analysis. Fungal diversity within communities (alpha diversity) was determined by analysis of OTUs and shown through estimators of the Shannon, Simpson, invSimpson and Fisher alpha indices. Observed and estimated richness was determined according to the following estimators: number of observations (OBS), Chao1 and ACE. Beta-diversity or diversity shared across sample communities was determined using Principal Coordinates Analysis (PCoA), as well as through redundancy analysis. Additionally, Venn diagrams were created in order to reflect shared diversity covered by the obtained OTUs. All figures were obtained using distance matrices for the genus taxonomic level. Supplementary Table S1, which shows the proportion of fungal communities, contains information about the relative abundances in all samples at the phylum (sheet 1), family (sheet 2), genus (sheet 3), and species (sheet 4) levels.

Data were deposited in the BioProject database (NCBI) as PRJNA553251^1^.

### *In vitro* Examination of Antifungal Activity of Autochthonous Bacterial Isolates on *Monilinia* spp.

The potential antagonistic activity of 67 bacterial isolates and 10 yeast isolates from the plum phyllosphere obtained in a parallel separate study (unpublished data) was tested on all 11 fungal isolates *in vitro*. The bacterial strains were cultured in Luria–Bertani broth and yeasts in yeast dextrose broth for 24 h at a temperature of 30°C. For the initial screening against all *Monilinia* spp., PDA plates were inoculated with four bacterial or yeast isolates aligned in rectangular form with a distance of 2.5 cm away from a 7-day-old fungal mycelial plug (5 mm in diameter). After 7 days of incubation at 25°C, antifungal activity was observed. The three most sensitive *Monilinia* isolates (M1, M8, and M13) and one most potent bacterial antagonist (P4/16_1) were selected for further screening of antagonistic activity. Antagonistic activity was quantified using a method of dual cultivation as described elsewhere (Dimkić et al., 2015). The mycelial plugs of each fungus were sampled from the periphery of 7-day-old cultures and plated on the PDA surface, about 25 mm from the center of each Petri dish. A broth culture of the tested bacteria (grown for 24 h in Luria broth at 30°C) was then streaked 3 cm away from the plugs of the test fungi on the same dish. Plates with only *Monilinia* spp. isolates were used as controls. All cultures were incubated for 7 days at 25°C. Effects on mycelial growth were evaluated by calculating the percent of growth inhibition, $P\,G\,I\%=\frac{100\,\left(K\,R-R\,1\right)}{K\,R}$, where KR represents the distance (measured in mm) from the point of inoculation to the colony margin on the control dishes, and R1 is the distance of fungal growth from the point of inoculation to the colony margin on the treated dishes in the direction of the antagonist. The experiments were repeated twice independently, with three replications for each fungus.

### Molecular Identification of the Antagonistic Bacterial Strain and Its Secondary Metabolites

Genomic DNA from P4/16_1 was isolated using the commercial ZR Fungal/Bacterial DNA MiniPrep^TM^ Kit according to the protocol of the manufacturer (Zymo Research, United States). The 16S rRNA gene was amplified using universal primers and the *gyrB* gene with *Pseudomonas*–specific sequences. Moreover, the distribution of antibiotic genes was tested using protocols described elsewhere. The oligonucleotide primers and their characteristics are listed in Table 1. For all PCR reactions, 30 cycles were used for amplification, except in the case of the *gyrB* gene, where 35 cycles were applied. All denaturation and elongation steps were performed at 95 and 72°C, respectively. All positive amplicons were purified using a column of the QIAquick PCR Purification Kit/250 (QIAGEN GmbH, Hilden, Germany) and sent for sequencing to the Eurofins sequencing service (Germany). The sequences were searched for homology in the GenBank database using the Blast search program for nucleotides of the NCBI.

**TABLE 1:** Characteristics of the primers used for PCR analysis.

**Targeted gene**	**Primer name**	**Primer sequence (5′-3′)**	**Tm (°C)**	**Ann. time (s)**	**Exp. size (bp)**	**References**
16S rRNA of small ribosomal subunit	UN16S-F	GAGAGTTTGATCCTGGC	51	30	1500	Dimkić et al., 2013
	UN16S-R	AGGAGGTGATCCAGCCG				
*gyrB* gene of gyrase B subunit	gyrB-F	MGGCGGYAAGTTCGATGACAAYTC	58	60	620	Sarkar and Guttman, 2004
	gyrB-R	TRATBKCAGTCARACCTTCRCGSGC				
*phcA* gene of phenazine-1- carboxylic acid	PhCA-F	TTGCCAAGCCTCGCTCCAAC	67	45	1150	Raaijmakers et al., 1997
	PhCA-R	CCGCGTTGTTCCTCGTTCAT				
*prnD* gene of pyrrolnitrin	PRN-F	GGGGCGGGCCGTGGTGATGGA	68	60	786	de Souza and Raaijmakers, 2003
	PRN-R	YCCCGCSGCCTGYCTGGTCTG				
*pltC* gene of pyoluteorin	PLTC1-F	AACAGATCGCCCCGGTACAGAACG	67	60	438	de Souza and Raaijmakers, 2003
	PLTC2-R	AGGCCCGGACACTCAAGAAACTCG				
*hcnBC* gene of HCN synthase	HCN-F	ACTGCCAGGGGCGGATGTGC	63	30	587	Ramette et al., 2003
	HCN-R	ACGATGTGCTCGGCGTAC				

Obtained 16S rRNA and *gyrB* sequences of P4/16_1 were used for concatenated sequences analysis. Sequences of reference strains were retrieved from GenBank database. Concatenated 16S rRNA and *gyrB* sequences of *P. aeruginosa* ATCC 15692 (GenBank accession: CP017149) were included as outgroup. Sequences were aligned by the CLUSTALW program integrated in BioEdit 7.2.6 software (BioEdit, RRID:SCR_007361). The concatenated dataset was used to construct dendrograms in MEGA 6 software (MEGA Software, RRID:SCR_000667) using Neighbor-joining methods. The percentage of replicate trees in which the associated taxa clustered together in the bootstrap test (1000 replicates) is shown next to the branches.

### Test for Phenazine-1-Carboxylic Acid Production, and Antifungal Metabolite Extraction

Testing of the P4/16_1 isolate’s ability to produce phenazine-1-carboxylic acid (PCA) was performed according to the method of Parejko et al. (2012). The isolate was grown overnight in Luria broth medium, with shaking at a rate of 180 rpm at a temperature of 25°C. Overnight culture was diluted to 10^5^ CFU/ml and 10 μL of suspension was spotted on PDA plates and tryptic soy agar (TSA) plates as a control. Results were recorded after incubation at 25°C for 2, 4, and 7 days and considered positive for PCA production if dark green crystals were observed within the isolate spot.

Antifungal metabolite extraction was performed following the procedure of Jain and Pandey (2016). Culture was grown for 4 days in a mineral salt medium at 25°C (composition in g/l: NaNO_3_ 3 g, MgSO_4_ ⋅ 7H_2_O 0.5 g, KCl 0.5 g, K_2_HPO_4_ 0.1 g, FeSO_4_ ⋅ 7H_2_O 0.01 g, yeast extract 5 g, glycerin 1%, pH 7.0). After incubation, the cell-free supernatant was acidified to pH 2.0 with concentrated HCL and extraction was performed twice with an equal volume of benzene. The benzene fraction was separated in a separating funnel, filtered through filter paper and evaporated to dryness on a rotary evaporator at 55°C. The dry crude extract was resuspended in methanol (at a final concentration of 10 mg/mL) for characterization and antifungal study.

### Production of Volatile Organic Compounds and Antifungal Activity of the Benzene Extract *in vitro*

Ability of the P4/16_1 isolate, initially originating from Požegača cultivars, to produce volatile organic compounds (VOC) was tested against three *M. laxa* isolates using the double plate technique as described by Giorgio et al. (2015). One PDA plate was inoculated with a mycelial plug, and doubly diluted overnight bacterial culture was spread in the other PDA plate, which was of equal size. The plates without lids were tightly sealed together face to face with Parafilm^®^ and incubated at 25°C for 2 weeks. A control for each fungus was also maintained. Mycelia diameters were measured, and the percent of inhibition was calculated.

The antifungal effect of the benzene extract against three *M. laxa* isolates was tested *in vitro*. A final volume of 100 μL of extract dissolved in methanol was spread on a PDA plate. A mycelial plug from the periphery of 7-day-old cultures was placed in the center of the PDA plate. The experiment was repeated twice independently, with three replications for each fungus. Inoculated plates were incubated at 25°C and diameter measurements were conducted after 7 days. The same formula mentioned above was used, and inhibition percentage of radial growth (PIRG%) were calculated. However, KR represents growth of the test fungus in the control, and R1 is growth of the test fungus in the presence of the inhibitory agent.

Data obtained were subjected to analysis of variance (one-way ANOVA). Separation of mean percentages of *in vitro* mycelial growth inhibition was analyzed using Tukey’s HSD (honest significant difference) test with a significance level of *p* < 0.05 in IBM SPSS Statistics, v. 20 (SPSS, Inc.) (SPSS, RRID:SCR_002865).

### Analysis of Bacterial Benzene Extract by LC/MS

Crude benzene extract was analyzed using a Thermo Fisher Scientific Accela ultrahigh-performance liquid chromatography (UHPLC) system coupled to a linear Ion Trap/OrbiTrap hybrid mass spectrometer (LTQ OrbiTrap MS). The mass detector was set to work in a negative ionization mode. A Syncronis C18 (100 × 2.1 mm, particle size 1.7 μm) analytical column was used for separation of aromatic compounds. The mobile phase consisted of (A) ultrapure water +0.2% formic acid (MS grade) and (B) acetonitrile (MS grade). A linear gradient program at a flow rate of 0.275 mL/min was used: 0.0–1.0 min 5% B, 1.0–16.0 min from 5 to 95% B, 16.0–16.1 min from 95 to 5% B, then 5% B for 6 min. Parameters of ion source and data-dependent settings were as previously described by Vasić et al. (2019). Full-scan analysis was employed for detection of the monoisotopic masses of deprotonated unknown compounds, while the MS^4^ experiment provided fragmentation pathways. The ChemDraw molecule editor program (version 12.0) was used as a reference library to calculate accurate mass of compounds of interest. Tentative identification of compounds of interest was achieved by high-resolution mass spectrometry (HRMS) and MS^n^ fragmentation.

### Microscopic Examination of Hyphal Morphology

Morpho-physiological changes in hyphae of three *M. laxa* isolates induced by benzene extract of the P4/16_1 isolate under co-culture growth conditions were observed using optical and scanning electron microscopy (SEM). For optical microscopy, samples were mounted in the standard mycological dye Lactophenol Cotton Blue and observed under a Zeiss AxioImager M1 microscope with AxioVision Release 4.6 software. Scanning electron microscopy was performed at the University of Belgrade, Faculty of Mining and Geology using a JEOL JSM-6610LV microscope with a W filament gun. Samples were gold-coated (*d* = 15 nm, ρ = 19.2 g/cm^3^) using a Leica EM SCD005 sputter coater. Secondary electron and back-scattered electron images were obtained at a 20 kV acceleration voltage in the high-vacuum mode (15–30 μPa in the sample chamber) with magnifications from 150× to 30,000×.

## Results

### Metagenomic Data and Fungal Diversity

The phylogenetic composition of fungal communities associated with *P. domestica* was analyzed using eight DNA samples isolated from four plum cultivars in two phenological stages [during early (May) and late (July) fruit maturation] by amplifying and sequencing the 3F–4R region of ITS2. The majority of sequences had an average sequence length of 525 bp. After trimming and quality filtering, 1,144,352 (May) and 1,066,790 (July) classifiable paired end sequence reads were retained (Supplementary Table S2).

Following the OTU-clustering and chimera-checking steps, total numbers of 2,310 and 3,363 OTUs were obtained in May and July, respectively. The number of OTUs shared by plums was 121 in May and 189 in July (Supplementary Figure S1). However, there was a difference between the two phenological stages with respect to the number of unique OTU sequences among cultivars. The number increased significantly in July for Rodna, Lepotica and Ranka cultivars For the Požegača cultivar, the situation was opposite. The total numbers of unique OTUs detected in Požegača, Rodna, Lepotica, and Ranka samples were 716, 740, 365, and 489, respectively, in May; and 762, 1,028, 711 and 862, respectively, in July.

**FIGURE 1:**
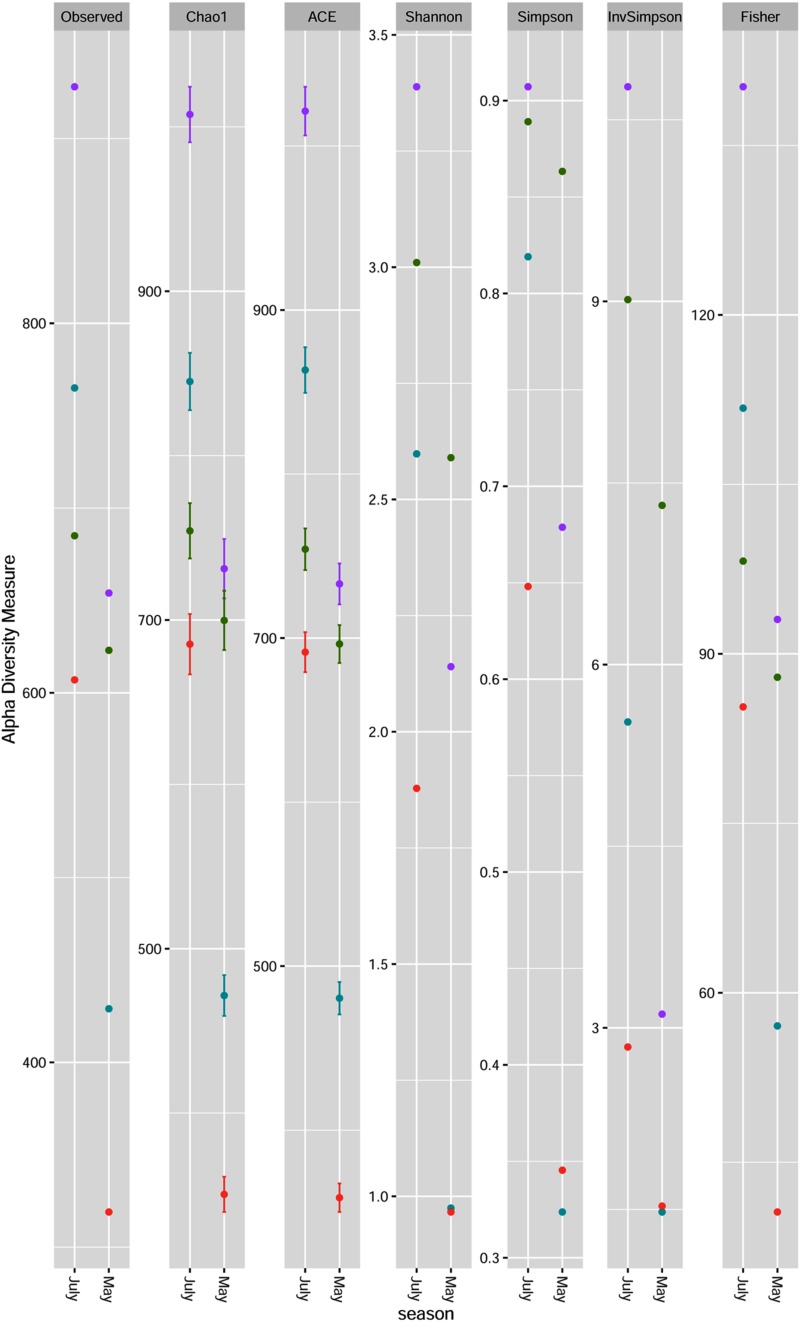
Fungal community richness and diversity of four plum cultivars [Lepotica (red dot •), Rodna (purple dot •), Požegača (green dot •) and Ranka (teal blue dot •)] at two phenological stages presented at the genus level. Richness is estimated by observed OTUs, Chao1 and ACE. Fungal diversity was evaluated by Shannon, Simpson, InvSimpson and Fisher.

The microbial richness and alpha diversity indices for each sample on the genus level are presented in Figure 1. Fungal communities were rich (especially in July) and diverse for each sample. All indices showed less diversity and evenness for all plum cultivars samples collected in May. High fungal diversity was observed for Požegača, relatively high diversity for Rodna and lower diversity for Ranka and Lepotica in May. The diversity was considerably higher in July, when the order of cultivars with respect to this index was as follows: Rodna > Požegača > Ranka > Lepotica. Alpha diversity indices estimated for the Požegača cultivar in May and July did not differ significantly. The differences between observed and estimated richness are in positive correlation according to the Chao1 and ACE indices and with observed OTU values, for all samples. The Chao1 and ACE indices indicated greater richness in samples collected in July compared to ones collected in May, the highest values being detected for Rodna and the lowest for Lepotica in both sampling periods. These results are in positive correlation with diversity values.

**FIGURE 2:**
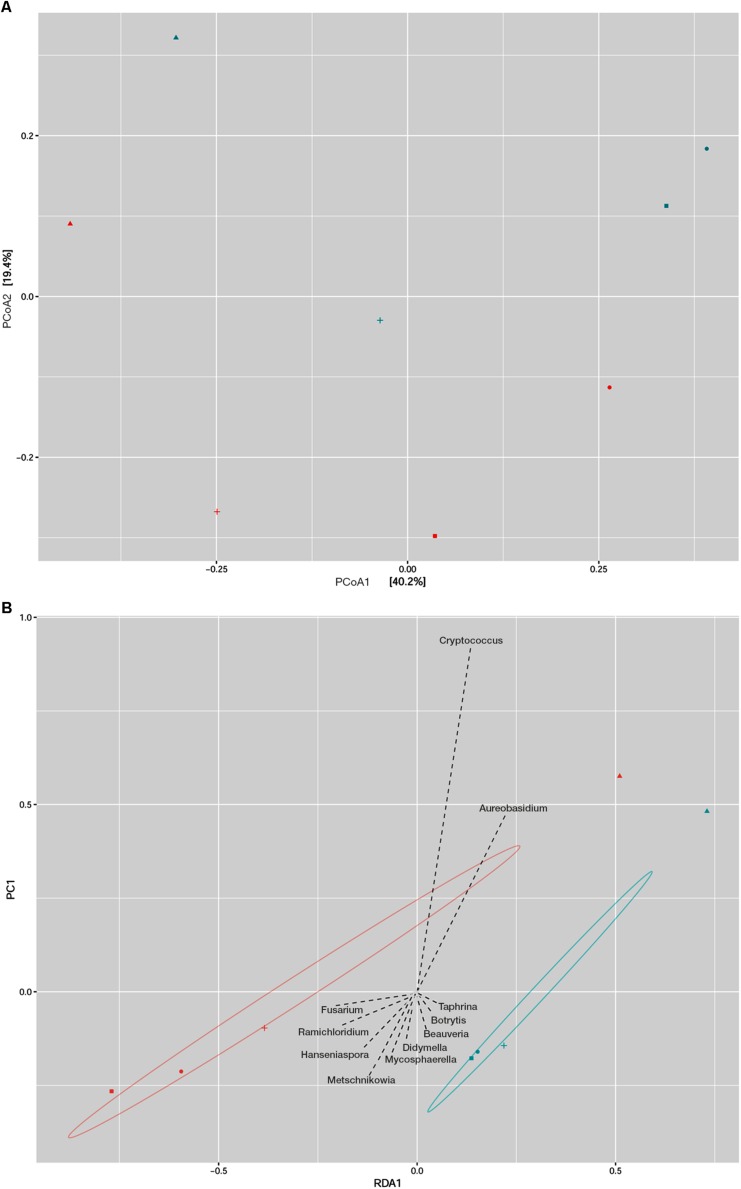
Beta diversity analysis of fungal communities in two phenological stages of four plum cultivars (Lepotica, Rodna, Požegača, and Ranka). Fungal communities from eight samples was compared by Principal coordinate analysis (PCoA) where the two axes represent 59.6% of the variation in the samples **(A)** and with Redundancy analysis (RDA) where are presented fungal genera as explanatory variables of fungal communities **(B)**. Plum cultivars are represented on plot with Lepotica (•), Požegača (▲), Ranka (■), and Rodna (+). Blue and red symbols represent color-coded samples from May and July, respectively.

Principal coordinate analysis (PCoA) and redundancy analysis were performed as a summary of beta diversity relationships of fungal communities among different plum cultivars. The PCoA analysis revealed a close association between the Ranka and Lepotica cultivars in both sampling periods, and they were clearly separated from all other samples (Figure 2A). First axis was able to explain 40.2% and second axis 19.4% of the variability of response variables. According to the main axis of disjunction (40.2%), the fungal communities of Rodna and Požegača in both phenological stages were grouped together and less dissimilar in comparison with the other two cultivars. Redundancy analysis (RDA) was used to identify fungal genera that significantly contributed to the structural difference between phyllosphere fungal communities of plum. On the RDA plot presented in Figure 2B, the RDA1 component separated cultivars according to sampling seasons, with the exception of Požegača, while the PC1 component separated Požegača from the other samples, with *Cryptococcus* and *Aureobasidium* as the dominant genera. A scatter plot revealed positive correlation with PCoA, indicating that autochthonous fungal communities depend on the season of collecting in the Ranka, Rodna, and Lepotica cultivars.

### Fungal Community Composition and Taxonomic Analysis

**FIGURE 3:**
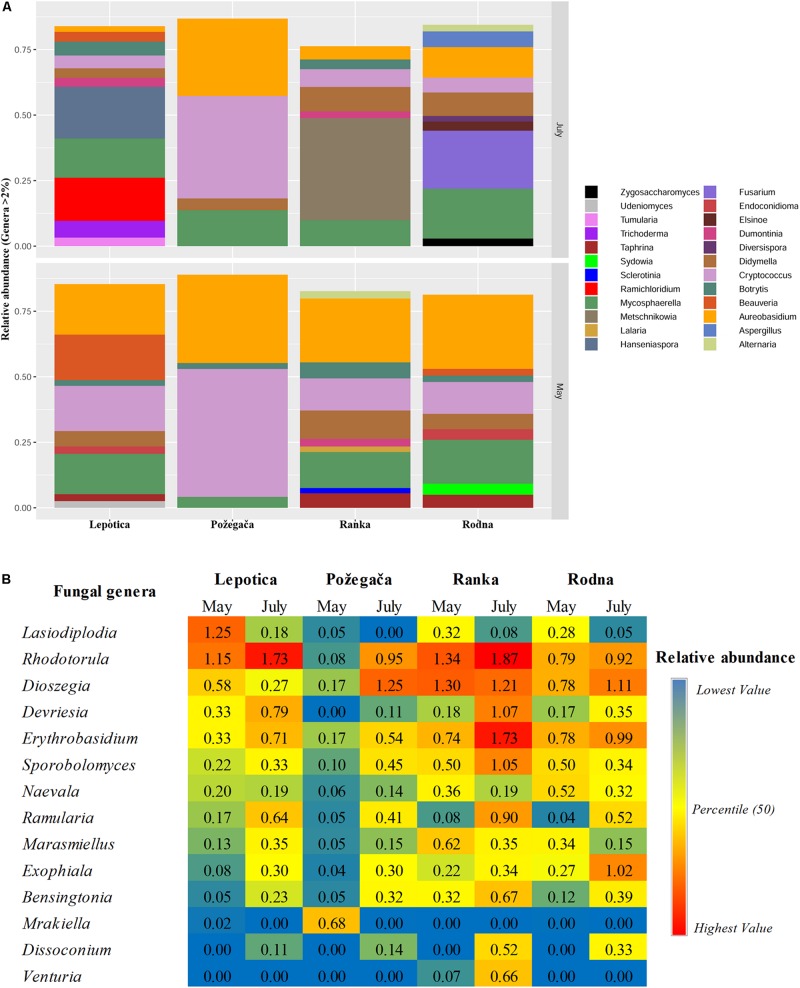
Relative abundance (RA) of fungal genera associated with four plum cultivars at two phenological stages (May and July). Genera with a total RA percentage above 2% **(A)** and between 0.5 and 2% **(B)** are presented.

In order to examine which fungal taxa are present, the representative sequence of each OTU was identified at different classification levels. The OTUs were aggregated on the phylum, family and genus levels, and those with a normalized abundance of over 2% in any sample were found and filtered (Supplementary Figures S2 and Figure 3). Analysis of ITS2 DNA region sequence data revealed that at the phylum level, Ascomycota was the most abundant in all samples, with a relative abundance (RA) of between 46% in Požegača in May and 89% in the Lepotica cultivar in July [Supplementary Table S1 (sheet 1) and Supplementary Figure S2]. Members of the phylum Basidiomycota were less represented, with an incidence of between 10 and 23%, except in the case of the Požegača cultivar, with RA values of 53 and 44% in May and July, respectively. Besides the two prevalent phyla, an RA abundancy threshold of 2% was reached only by the phylum Glomeromycota in the Rodna cultivar in July. A similar situation was noticed on the family level, where some families were considerably more abundant in some samples than in others. The most abundant families belonging to the phylum Ascomycota were Mycosphaerellaceae [with RA in the range of from 4.21% (Požegača) to 31.77% (Lepotica)] and Dothioraceae [with RA in the range of from 2.44% (Lepotica) to 33.44% (Požegača)], followed by Sclerotiniacae [from 0.2% (Požegača) to 14.6% (Ranka)] and Taphrinaceae [from 0.51% (Lepotica) to 7.05% (Ranka)]. High abundance of an incertae sedis taxon within the phylum Basidiomycota and order Tremellales was detected in Požegača in May (49.13%) and July (40.69%), while in the other cultivars its presence varied between 5.77 and 17.74%. Conspicuous differences were scored for the Rodna cultivar, where in May, Dothioraceae and Pseudeurotiaceae dominated among the other families, while in July, the families Nectriaceae (21.5%), Trichocomaceae (5.8%), Elsinoaceae (3.7%), and Saccharomycetaceae (2.8%) were notably present as well. Moreover, in July Saccharomycodaceae (18.8%) and Hypocreaceae (6.1%) were notably present on the Lepotica cultivar and Metschnikowiaceae (37.5%) on the Ranka cultivar.

**FIGURE 4:**
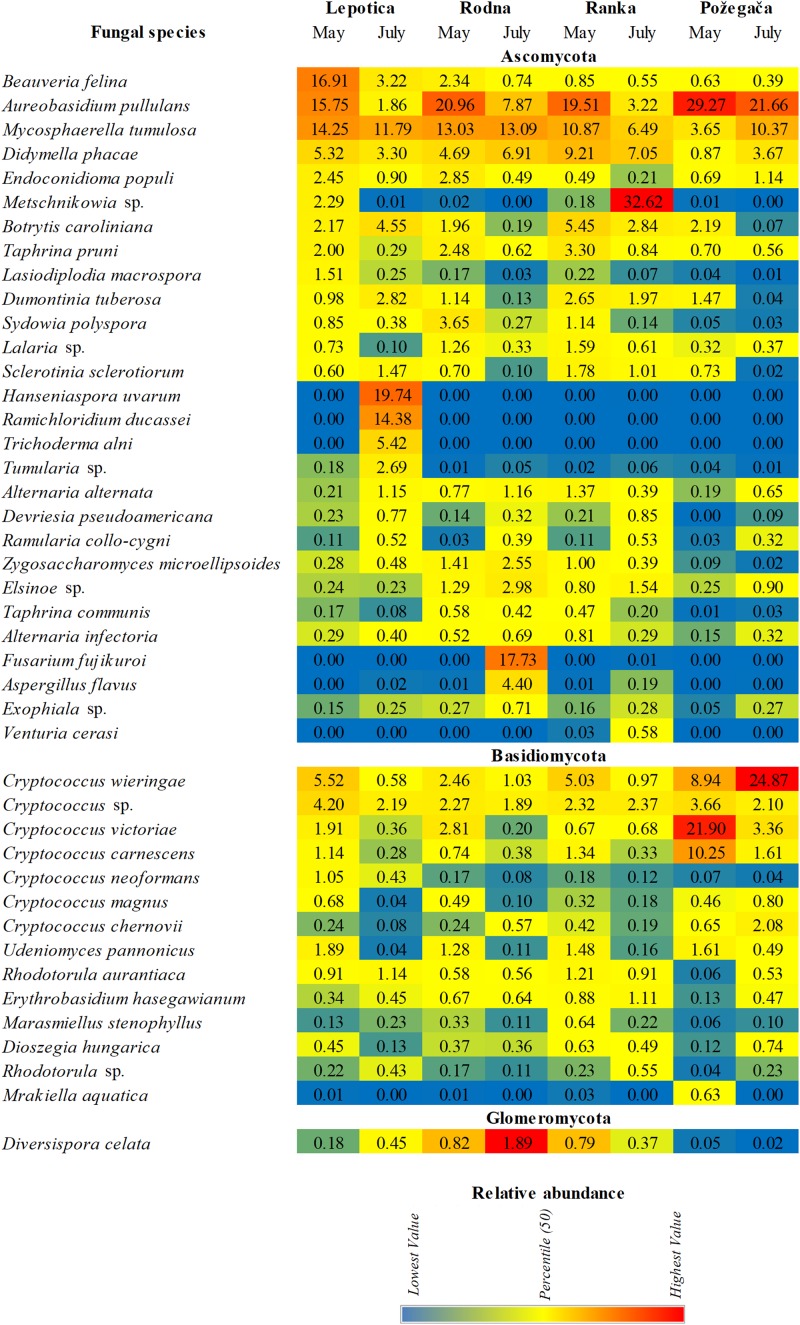
Relative abundance (RA) of fungal species associated with four plum cultivars at two phenological stages (May and July).

The relative abundance of detected genera on four cultivars is presented in Figure 3. A large number of taxa were identified, but only a few genera accounted for most of the reads. The most abundantly present genus on all plum cultivars in May was *Aureobasidium* (phylum Ascomycota), with RA values ranging from 19.27 to 33.69%, followed by *Cryptococcus* (phylum Basidiomycota), with RA values from 4.8 to 48.80%. In July, different genera were dominant on particular cultivars: *Metschnikowia*, with 38.95%, on Ranka *Fusarium*, with 22.12%, on Rodna; and *Hanseniaspora*, with 19.77%, on Lepotica. The predominant genus on Požegača was *Cryptococcus*, with RA of 48.80% in May and 39.10% in July. Also, according to RA values, the genus *Mycosphaerella* was always detected among the first four genera (4.19 – 16.77%) on all plum cultivars. It is interesting to note that stone fruit pathogens such as *Didymella* (10.8%), *Botrytis* (6.1%), *Taphrina* (5.6%), *Alternaria* (2.8%), and *Sclerotinia* (2%) were dominant on the Ranka cultivar in May, while in July their presence was lower. Besides these most abundant genera detected on cultivars, Figure 3B presents all genera with RA values between 0.5 and 2%. It was noticed that in July on Ranka, the genera *Rhodotorula, Erythrobasidium, Sporobolomyces, Ramularia, Bensingtonia*, and *Venturia* were dominant on the given cultivar, as well as *Exophiala* on Rodna. Other genera of fungal stone fruit pathogens such as *Monilinia*, *Cladosporium*, and *Penicillium* were represented with RA values of less than 0.1%. In addition to differences in overall diversity, different fungal species dominate communities of the four studied cultivars in percentages similar to those recorded on the genus level (Figure 4). These results are in positive correlation with the spatial dynamics previously observed through all of the mentioned taxa. In the phylum Ascomycota, *Aureobasidium pullulans, Mycosphaerella tumulosa* and *Didymella phacae* were most abundant throughout both phenological stages on all cultivars. The entomopathogenic fungi *Beauveria felina* and *Endoconidioma populi* were notably present on Lepotica in May, while *Hanseniaspora uvarum*, *Ramichloridium ducassei* and *Trichoderma alni* were recorded during July. Also, in July on the Rodna cultivar, *Fusarium fujikuroi*, *Aspergillus flavus*, and *Alternaria alternata* were scored with higher percentages than those recorded for other species. Within the phylum Basidiomycota, the genus *Cryptococcus* was quite heterogenetic, with *C. victoriae*, *C. wieringae* and *C. carnescens* as the most abundant species among others, especially on Požegača in both phenological stages. The most prevalent species within the phylum Glomeromycota was *Diversis poracelata*.

### Identification of *Monilinia* spp.

Based on morphological characteristics, 11 isolates sampled from four plum cultivars were preliminarily identified as *Monilinia laxa*. After 7 days of incubation on PDA at 25°C in the dark, all fungal isolates were white to light-gray with concentric rings of mycelium and lobbed margins, rosetted, without sporulation rings. Molecular identification of *Monilinia* isolates was done by amplifying the internal transcribed spacer between genes for the 5.8S and 28S ribosome subunits, yielding DNA fragments of 550 and 900 bp, respectively. The obtained isolates were identified as *M. laxa*. The sequences of amplified DNA fragments from all 11 isolates with the ITS1/ITS4 set of primers had 100% nucleotide similarity with *M. laxa* isolates from GenBank. The DNA fragments amplified with the ITS3/TW13 set of primers showed sequence identity with *M. laxa* with only one nucleotide difference for 10 isolates. For both amplified regions, all isolates from this study were identical to each other. A neighbor-joining tree was reconstructed using sequences of *M. laxa* isolates that included ITS sequences of *Monilinia* spp. strains from GenBank, with *S. sclerotiorum* as an outgroup. Due to poor sequence quality, isolate M12 was excluded from the ITS3/TW13 tree. In both phylogenetic trees (Supplementary Figure S3), all sequences from this study were grouped in one cluster with *M. laxa* sequences.

### Identification and Characterization of an Antagonistic Bacterial Strain

Isolate P4/16_1 was first identified by sequencing of the 16S rRNA gene. Our BLAST database search revealed three species with the same scores: *Pseudomonas synxantha*, *Pseudomonas mucidolens*, and *Pseudomonas libanensis*. Afterward, the *gyrB* region was amplified and the concatenated phylogenetic tree was made from the 16S rRNA and *gyr*B sequences (Supplementary Figure S4) that confirmed isolate P4/16_1 as *P. synxantha*.

Results of screening for genes coding antibiotics were negative for pyrrolnitrin, pyoluteorin and hydrogen cyanide. However, amplification of the gene coding for phenazine-1-carboxylic acid gave one distinct DNA band with a length of about 1150 bp. Sequencing results confirmed that amplicon as belonging to the putative phenazine biosynthesis protein of *P. synxantha* LBUM223 (max/total score: 2065; query cover: 100%; E value: 0.0; ident.: 100%).

### Chemical Analysis of the Crude Benzene Extract

**FIGURE 5:**
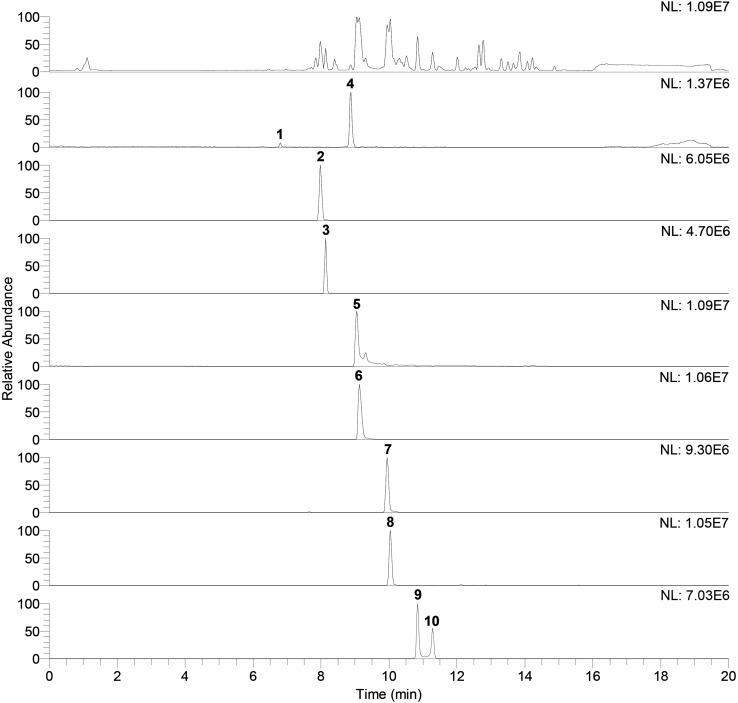
Base peak chromatogram of antagonist benzene extract (top of the figure) and extracted ion chromatograms. Peaks that belong to identified compound are labeled with corresponding compounds number. Retention times (tR) and exact mass data in the negative ionization mode ([M–H]–), for identified compounds, are given in the Table 2.

**FIGURE 6:**
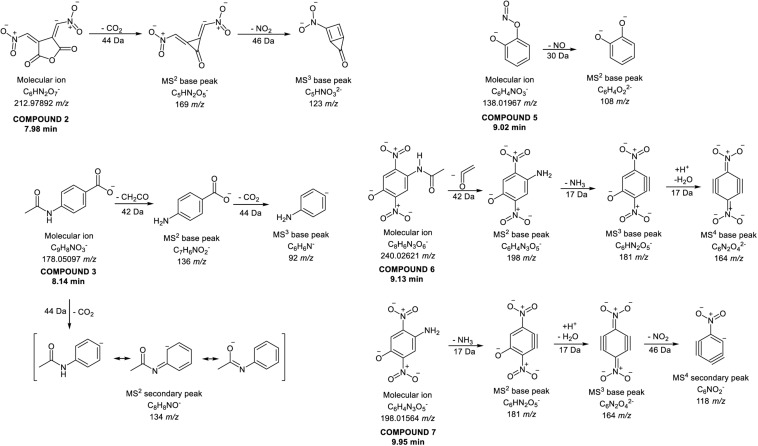
Proposed fragmentation patterns of some identified compounds in the crude benzene extract.

Identification of compounds in the crude benzene extract was performed by an exact mass search of deprotonated molecules ([M–H]^–^) and corresponding MS^4^ fragmentation. A base peak chromatogram of the obtained benzene extract is shown in the top part of Figure 5. Extracted ion chromatograms of identified compounds are shown below the main chromatogram, and their retention times (*t*_R_, min), molecular formulas ([M–H]^–^), calculated and exact masses (*m/z*) and mean mass accuracy (Δ ppm) are given in Table 2, together with major MS^2^, MS^3^ and MS^4^ fragment ions. With the same molecular ion at 137 *m/z*, compounds **1** and **4** (at 6.80 and 8.88 min) were identified as structural isomers of hydroxybenzoic acid [2-hydroxybenzoic acid (salicylic acid) and 4-hydroxybenzoic acid]. Both compounds gave an MS^2^ base peak at 93 *m/z*, generated by loss of CO_2_ (44 Da). Identity of the compound under the first signal (at 6.80 min) was confirmed using the available standard of 4-hydroxybenzoic acid, and the second peak (at 8.88 min) showed the signal of salicylic acid (2-hydroxybenzoic acid) (Marengo et al., 2001). The structures of compounds **2**, **3**, **5**, **6,** and **7** were deduced by examination of their exact masses and corresponding MS^n^ fragmentations (Table 2). Fragmentation patterns and the proposed structures of fragment ions of these compounds are shown in Figure 6. Compounds **8**, **9,** and **10** were not identified, but their MS data are nevertheless shown in Table 2.

**TABLE 2:** LC/MS data of compounds identified in crude benzene extract.

**Peak No**	***t*_R_, min**	**Molecular formula, [M–H]^–^**	**Calculated mass, [M–H]^–^**	**Observed mass, [M–H]^–^**	**Δ (ppm)**	**MS^2^ Fragments (% Base Peak)**	**MS^3^ Fragments (% Base Peak)**	**MS^4^ Fragments (% Base Peak)**	**Name**
1	6.80	C_7_H_5_O_3_^–^	137.02442	137.02451	–0.66	109(5), **93**(100)	n.d.	n.d.	**4-hydroxybenzoic acid**
2	7.98	C_6_HN_2_O_7_^–^	212.97892	212.97899	–0.33	**169**(100)	138(20), **123**(100), 115(10), 107(25)	n.d.	**(3*Z*,4*Z*)-3,4-bis(nitromethylene) dihydrofuran-2,5-dione**
3	8.14	C_9_H_8_NO_3_^–^	178.05097	178.05074	1.29	**136**(100), 134(30)	**92**(100)	n.d.	**4-acetamidobenzoic acid**
4	8.88	C_7_H_5_O_3_^–^	137.02442	137.02438	0.29	109(5), **93**(100)	n.d.	n.d.	**hydroxybenzoic acid**
5	9.02	C_6_H_4_NO_3_^–^	138.01967	138.01942	1.81	**108**(100)	n.d.	n.d.	**2-hydroxyphenyl nitrite**
6	9.13	C_8_H_6_N_3_O_6_^–^	240.02621	240.02548	3.04	**198**(100)	**181**(100), 168(90), 151(5), 138(45), 89(10)	**164**(100), 151(15), 134(15), 120(40), 106(10)	***N*-(4-hydroxy-2,5-dinitrophenyl) acetamide**
7	9.95	C_6_H_4_N_3_O_5_^–^	198.01564	198.01503	3.08	**181**(100), 168(85), 153(20), 138(45), 113(15)	**164**(100), 153(10), 134(35), 120(30), 106(10)	118(45), 109(10), 103(20), 93(15), **65**(100)	**4-amino-2,5-dinitrophenol**
8	10.04	C_12_H_6_N_3_O_3_^–^	240.04146	240.04048	4.08	**210**(100)	**182**(100), 166(10)	**154**(100)	**Not identified**
9	10.84	C_13_H_8_N_2_O_2_^–^	224.05967	224.05827	6.25	195(5), 181(55), **180**(100)	**150**(100), 136(20), 135(40), 123(85)	n.d.	**Not identified**
10	11.27	C_13_H_8_N_2_O_2_^–^	224.05967	224.05872	4.24	181(35), **180**(100)	150(75), 134(15), 123(70), 117(70), **109**(100)	n.d.	**Not identified**

*Bold font represent basic ions (ions that have a relative intensity 100%).*

### Antifungal Activity

**FIGURE 7:**
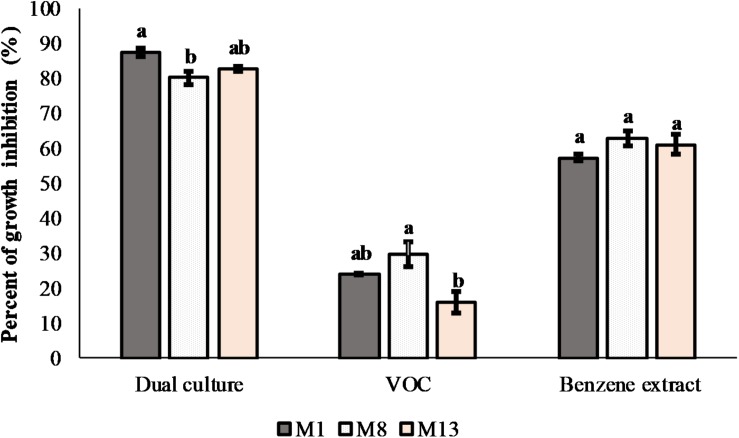
Antifungal effects of *P. synxantha* isolate P4/16_1 in dual culture with production of volatile organic compounds and in interaction with benzene extract *in vitro* against three *Monilinia laxa* isolates. Mean values of percent of inhibition (*n* = 3) of fungal growth with standard errors are shown. Values followed by the same letter on the histogram columns were not significantly different (*P* < 0.05) according to Tukey’s HSD test.

Screening of 67 bacterial and 10 yeast isolates from the plum phyllosphere for antagonistic activity against 11 *Monilinia* isolates was initially done. Moderate activity was detected for 26 bacterial and nine yeast isolates. Such activity limited further mycelial growth of most *Monilinia* isolates at the line of the contact. One isolate (P4/16_1), identified as *P. synxantha*, had significant inhibitory activity, with a large clear zone formed against three *M. laxa* isolates. Subsequently, the *P. synxantha* isolate and three *Monilinia* isolates (M1, M8, and M13) were chosen for further testing in dual culture assays. The 24-h overnight culture of P4/16_1 inhibited mycelial growth in the range of from 80 to 87.5%. The crude benzene extract exhibited 57.2–63.04% radial inhibition of mycelial growth, while the VOC that P4/16_1 produced in sealed PDA plates decreased growth of the tested *M. laxa* between 15.94 and 29.58% after 14 days of incubation (Figure 7).

**FIGURE 8:**
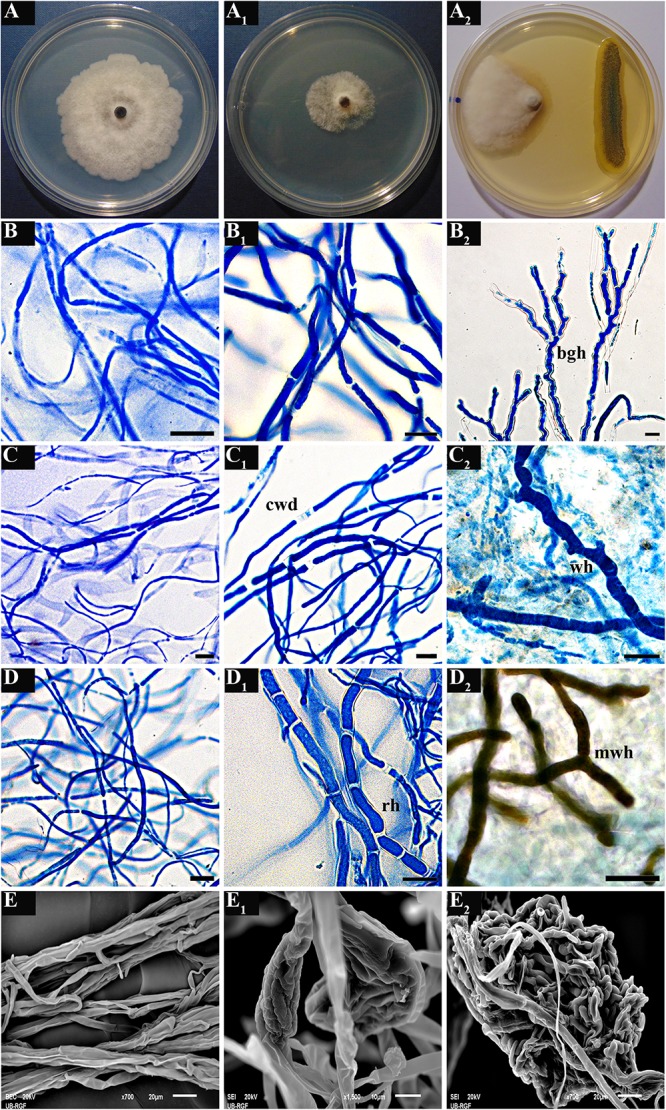
Morpho-physiological changes in hyphae of three *M. laxa* isolates (M1, M8, and M13) induced by crude benzene extract of *P. synxantha* P4/16_1 **(B_1_,C_1_,D_1_)** and under co-culture growth conditions **(B_2_,C_2_,D_2_)** as observed with optical microscope. Changes in hyphae of *M. laxa* M8 observed under an SEM: normal hyphal morphology in control culture **(E)**; hyphal proliferation induced by benzene extract **(E_1_)**; mass of intertwined hyphae formed in co-culture **(E_2_)**. Typical hyphal morphology of *Monilinia* isolates from growth controls under a light microscope **(B–D)** or using an SEM **(E)** and appearance of the mycelium on a plate as a control **(A)** are shown, as well as culture treated with benzene extract **(A_1_)** and dual culture **(A_2_)**. cwd, disintegration of cell wall; rh, robust hyphae with recurrent septation; bgh, branched geniculate hyphae; wh, conspicuous “worm-like” hyphae in mycelial mass; mwh, melanized “worm-like” hyphae. Scale bar = 20 μm.

Treatment of *M. laxa* with the crude benzene extract of *P. synxantha* P4/16_1, as well as co-culturing of *M. laxa* and *P. synxantha*, resulted in induction of various morpho-physiological changes in hyphae of the tested fungal isolates (Figure 8). Some of the prevalent alterations included frequent disintegration of the cell wall along hyphal segments, formation of highly septate robust hyphae intertwined with hyphae of normal morphology and appearance of highly branched geniculate hyphae (Figure 8B_1_). One-week co-culturing of *M. laxa* M8 and *P. synxantha* P4/16_1 resulted in the formation of conspicuous hyaline “worm-like” hyphae immersed in the mycelial mass (Figure 8C_2_). In the case of *M. laxa* M13, a melanized variation of the same hyphal morphology appeared along the margin of the colony facing the bacterial growth (Figure 8D_2_). Additionally, hyphal proliferation of isolate M8 was induced by the benzene extract (Figure 8E_1_), and a large mass of intertwined hyphae formed in the co-culture (Figure 8E_2_), facts that were confirmed using scanning electron microscopy.

## Discussion

In the present study, the diversity of fungi associated with plum leaves and fruits, was investigated at different phenological stages in order to characterize fungal communities on four *Prunus domestica* cultivars in an orchard not treated with pesticides. Rich fungal communities were identified on the four studied plum cultivars. The composition and diversity of fungal communities as estimated by alpha and beta diversity varied between cultivars and sampling seasons. These results are in accordance with reports, which indicate strong seasonal influence on richness and diversity of the microbial phyllosphere community on leaves of other plants (Penuelas et al., 2012; Haas et al., 2018). Within the phylum Ascomycota, the most abundant families on plums were Mycosphaerellaceae and Dothioraceae, followed by Sclerotiniacae and Taphrinaceae, all with important pathogenic members, some of them mainly associated with stone fruits, as reported earlier (Cannon and Kirk, 2007).

The fungal communities in the phyllosphere of four plum cultivars were characterized by a high prevalence of yeasts. At the early phenological stage in May, *Aureobasidium pullulans* was the most abundant species among the cultivars. *Cryptococcus* was also abundantly presented in all cultivars in spring samples with different species. Their presence was constant on Požegača cultivar in both sampling seasons, and their high prevalence was a major contributor to the difference from the other cultivars. These two genera have been recognized as typical constituents of the yeast community in the phyllosphere. Studies with the cultivation approach reported the presence of these genera throughout maturation of nectarines and plums (Janisiewicz et al., 2010, 2014). The use of the metagenomic approach in assessing microbial composition of the phyllosphere of organic strawberries revealed *Cryptococcus* as the most abundant fungus (Sylla et al., 2013). *Aureobasidium* was also one of the most abundant genera on grapes and olive leaves and flowers (Pinto et al., 2014; Abdelfattah et al., 2015). Members of the indicated genera have been reported to be effective biocontrol agents against a number of post-harvest diseases of various fruits, and some commercially available biocontrol products are based on them (Janisiewicz et al., 2014). Interestingly, *Metschnikowia* sp. had the highest RA value only on the Ranka cultivar in July. Very low RA values were detected on the other cultivars. That is in agreement with earlier studies (Janisiewicz et al., 2014), where *Metschnikowia* species were not isolated from plums and only sporadically from nectarines. The high prevalence of *Metschnikowia* sp., sugar-loving yeasts, on the Ranka cultivar may be caused by juices diffusing from a damaged surface of mature fruit (Barata et al., 2012), considering that this cultivar had the earliest time of maturation among the analyzed cultivars. Several *Metschnikowia* taxa, species such as *M. pulcherrima*, *M*. *fructicola*, and *M*. *andauensis*, were identified as biocontrol agents against *P. digitatum, P. expansum*, and *B. cinerea* (Janisiewicz et al., 2001; Manso and Nunes, 2011). Another yeast, *Hanseniaspora uvarum*, was detected only on the Lepotica cultivar in July, when fruits were in the mature phase. It has been frequently found on the surface of different fruits such as grapes, strawberries and apples (Santo et al., 2012; Graça et al., 2015), with a colonizing period in the more mature phases (Janisiewicz et al., 2010). It was noticed that this species has the ability to produce volatile thiols, in particular 4-mercapto-4-methyl-2-pentanone, 3-mercaptohexan-1-ol and 3-mercaptohexyl acetate, which are some of the most potent aroma compounds of alcoholic beverages (Dionísio et al., 2012). Microbial communities associated with plum are relevant for the fermentation process in production of alcoholic brandies. The non-*Saccharomyces* yeasts *Aureobasidium* and *Cryptococcus*, but also *Metschnikowia* and *Hanseniaspora*, play an important role in spontaneous alcoholic fermentations and in enhancing the composition and aroma of alcoholic beverages (Gschaedler, 2017). Serbia holds first place in Europe, with 158.000 ha under plum trees and with total production of 425 441 t of plums for the period 2013 – 2017, which places Serbia third among the top 10 plum producers according to FAOSTAT (2017) (Food and Agriculture Organization of the United Nations). Since most of the annual plum yield in Serbia is used for production of the specific regional brandy slivovitz, knowledge of the yeast microbiota could be of great significance. The best-known native plum cultivars in Serbia are Požegača and Ranka, which possess the most suitable characteristics for making high-quality brandies (Matković, 2015), but due to the constant development of fungal diseases, their numbers have been declining over the years.

The genus *Mycosphaerella* was constantly present on all cultivars in both seasons. Also, the cosmopolitan genera *Fusarium* and *Aspergillus*, were detected with high prevalence only on Rodna in July. This fact is quite important, since these two genera are comprised with pathogenic members well-known for their pathogenicity and mycotoxin production, and the Rodna cultivar is the most profitable variety in the Serbian product assortment of today (Matković, 2015). Likewise, the species *Ramichloridium ducassei* was also found in high percentages in the economically important Lepotica graft cultivar during July. The first record of this newly characterized pathogen was on banana leaves (Shivas et al., 2011). To the best of our knowledge, this is the first report indicating the presence of this species in the plum phyllosphere.

During the earlier phenological stage, the Ranka cultivar showed an increased presence of some of the most important stone fruit pathogens, species such as *Botrytis caroliniana*, *Taphrina pruni*, *Sclerotinia sclerotiorum*, and *Alternaria alternata*. However, it was noticed that their presence rapidly decreased parallel with increase in the presence of *Metschnikowia* yeasts, species of which were identified as biocontrol agents earlier (Janisiewicz et al., 2001; Manso and Nunes, 2011). Also detected were species belonging to a wide variety of other genera. Their presence and abundance varied between the cultivars and accounted for differences in the composition of fungal diversity on the four studied plum cultivars. Like those recorded in our research, differences between cultivars with respect to their microbiota were also noted elsewhere (Materatski et al., 2019).

The most important plum pathogen, *Monilinia* spp., was detected in a very low percentage, which coincides with our ability to cultivate only 11 isolates during two sampling periods on all four cultivars. The low abundance could be explained by conditions that were not favorable for germination of *Monilinia*, considering that the year 2016 was with very high temperatures and little precipitation (RHMZ, 2017).

Among different pathogens causing disease on stone fruits, we focused on brown rot-causing *M. laxa* as the most devastating in Serbia, with a presence of 89% in orchards affected by brown rot (Hrustić et al., 2015). Eleven isolates with morphological characteristics corresponding to *M. laxa* were confirmed as *M. laxa* by sequencing of the ITS region. For *Monilinia* spp., the ITS region is highly conserved and there are only a few nucleotide differences among these species, but they are sufficient to distinguish *M. laxa*, *M. fructigena*, and *M. fructicola* from stone fruit orchards in Serbia (Hrustić et al., 2015). Alongside *M. laxa*, isolates of *M. fructigena* were also significantly present in the genus *Monilinia* in most European countries (Petróczy et al., 2012; Poniatowska et al., 2013). Although *M. laxa* has long been known as a cause of brown rot of stone fruit in Europe, in the past two decades it was reported in other regions of the world as well, including Brazil, the United States, China, and Iran (Lino et al., 2016).

Considering the importance of *M. laxa* as a causative agent of devastating brown rot on plums, we aimed to find a potential candidate that will exhibit antifungal activity against brown rot causing fungi from the plum microbiota in the investigated orchard. To achieve this, we isolated the culturable diversity of microorganisms (bacteria, yeasts, and fungi) from the same samples used for metabarcoding (data not shown). Indigenous bacterial and yeast isolates from the phyllosphere of the analyzed four plum cultivars were tested *in vitro* for their antagonistic potential against *M. laxa*. The initial screening revealed that some isolates had the ability to inhibit mycelial growth on contact. For further testing, P4/16_1 was selected and identified as *P. synxantha* with the genetic potential to synthesize PCA. It was found that *P. synxantha* P4/16_1 exerted intensive inhibition at a distance away from mycelial plug. Pseudomonads are known for producing a wide variety of antimicrobial agents, compounds such as pyoluteorin, phenazines, pyrrolnitrin, cyclic lipopeptides and 2,4-diacetylphloroglucinol (DAPG) (Müller et al., 2016). In further investigation, we focused on phenazines, since the potential presence of a phenazine product was identified in the PCA test on a PDA plate. The *P. synxatha* P4/16_1 isolate exhibited expressed morphological changes, with formation of dark green crystals during growth on PDA plates, indicating that the isolate had the ability to produce phenazine carboxylic acid, according to Parejko et al. (2012). To be specific, dark green crystals were evident on P4/16_1 after 48 h of incubation. Accordingly, P4/16_1 culture growing 5 days in a minimal salt medium at 25°C was subjected to benzene extraction, and the benzene fraction was further tested for antifungal activity against three *M. laxa* isolates. The benzene extract dissolved in methanol showed moderate activity, with PGI% lower than the inhibitory activity obtained in the case of dual culture. It was also confirmed with the aid of optical microscopy and SEM that the effect of *P. synxantha* P4/16_1 in dual culture on *M. laxa* mycelia was different from that of the benzene extract, e.g., more pronounced changes of hyphal morphology were observed when they were growing together. Since no contacts between the bacterial and fungal colonies were established, melanization of the cell wall was presumably caused by a diffusible and soluble bacterial product other than that extracted by benzene. Similar results, with either diffusion of melanin on agar plates and/or melanization of fungal cells, were obtained by several authors: Machado et al. (2010a, b) in co-cultures of *Aspergillus niger*, *A. alternata* and *Fonsecaea pedrosoi* with *Bacillus subtilis*; and Frases et al. (2007) after co-culturing *Cryptococcus neoformans* with *Klebsiella aerogenes.* The observed melanization of the *M. laxa* hyphal cell wall is a typical stress response to adverse environmental conditions, known to be induced by several factors: microbial antagonism (as a way to increase antifungal resistance), host defense mechanisms, predation by amoebae, extreme temperatures, UV radiation, hydrolytic enzymes, oxidative radicals, metal toxicity (Fogarty and Tobin, 1996; Henson et al., 1999; Nosanchuk and Casadevall, 2003). In regard to bacteria, homogentisic acid, an intermediate in their catabolism, is capable of inducing the melanogenesis pathway in fungi (Frases et al., 2007).

The most commonly identified phenazine derivatives produced by *Pseudomonas* spp. are pyocyanin, PCA and a number of hydroxy-phenazines (Chin-A-Woeng et al., 2003; Garrido-Sanz et al., 2017). We performed benzene extraction from P4/16_1 culture grown according to conditions described in the literature (Jain and Pandey, 2016), assuming that the antimicrobial activity of P4/16_1 was due to PCA. However, chemical analysis revealed isomers of hydroxybenzoic acid and phenazine derivatives (compounds **8-10**). The ability of salicylic acid to inhibit a wide spectrum of fungal pathogens was shown in various studies (El-Mohamedy et al., 2013; Panahirad et al., 2013; da Rocha Neto et al., 2015). Amborabé et al. (2002) reported antifungal activities of a number of benzoic acid derivatives against *Eutypa lata*. From the peak chromatogram, it can be seen that many other compounds are also present in the crude benzene extract, and they might contribute to the extract’s observed activity. However, their identification and structural assignment were difficult due to unusual HRMS data suggesting an increased number of nitrogen atoms and lack of corresponding data in the literature. From the data provided in Table 2, it could be suggested that compounds **9** and **10** are structural isomers, since both compounds gave an MS^2^ base peak at 180 *m/z*, and that compounds **8**-**10** contain a phenazine type of heterocyclic system. As earlier studies have shown that *Pseudomonas* sp. produced phenazine-1-carboxylic acid (El-Sayed et al., 2008; Jain and Pandey, 2016), and in the present research we showed, by identification of the gene for production of PCA, that *P. synxantha* has the potential to synthetize PCA. Proper identification of compounds **8**–**10** requires preparative isolation and characterization using additional instrumental techniques, above all, advanced NMR methods. The explanation for absence of identification lies in conditions of the HPLC experiments. The HPLC-MS analysis was performed under basic conditions where decarboxylation of phenazine-1-carboxylic acid probably occurred.

Since pseudomonads are also known to produce volatile compounds, we tested the ability of *P. synxantha* P4/16_1 to produce VOCs *in vitro*. The results showed that P4/16_1 volatiles were able to moderately inhibit fungal growth. We hypothesized that the overall antifungal activity of this isolate was partly due to the production of VOCs. Other studies reported that VOCs of fluorescent pseudomonads had antifungal activity against *S. sclerotiorum* and *B. cinerea* (Giorgio et al., 2015; Hernández-León et al., 2015).

This is the first report of native fungal communities of the *P. domestica* phyllosphere obtained via the metabarcoding approach. Our study revealed high abundance of yeasts on plum cultivars, and important plum pathogens in low numbers. The metabarcoding protocols are already recognized as an investigative tool needed to provide foundation data for a more holistic use in control strategies. That kind of strategy would be based on populations of indigenous microbial antagonists as a new approach in management of plant pathogens (Abdelfattah et al., 2018). To the best of our knowledge, we report for the first time, the isolation of *P. synxantha* with antifungal potential against *M. laxa* under *in vitro* conditions. This isolate showed antagonistic activity by producing diffusible antifungal compounds and volatile organic compounds with antifungal activity against brown rot-causing fungi. We consider that as promising starting point to extend our research to potential use in biocontrol. Biocontrol agents have been used in agriculture for management of fungal and bacterial diseases, but there is little information about their impact on the resident microbiota. Future studies will consider treatment of the plum phyllosphere at the early and late phenological phase of fruit development with *P. synxantha* P4/16_1 in order to compare the treated plum phyllosphere with the untreated one as a way of determining whether changes occur in composition of the community, with an emphasis on changes in the presence of pathogens, especially *M. laxa*. In addition, capacity for postharvest application, in form of pure culture or active metabolite, would be pursued. Moreover, other isolates from plum phyllosphere, especially yeast, will be tested to include other potential biocontrol mechanisms that we did not monitor in this research.

## Data Availability Statement

The metagenomics datasets generated for this study can be found in the NCBI Sequence Read Archive as BioProject PRJNA553251 [https://www.ncbi.nlm.nih.gov/sra/PRJNA553251].

## Author Contributions

TJ, ID, NU, ML, DO, UG, SS, and TB conceived and designed the experiments, analyzed and interpreted the data, and wrote, critically revised and approved the final version of the manuscript. TJ, ID, NU, DO, and UG completed the experiments and collected the data.

## Conflict of Interest

The authors declare that the research was conducted in the absence of any commercial or financial relationships that could be construed as a potential conflict of interest.

## Footnotes

1 https://www.ncbi.nlm.nih.gov/sra/PRJNA553251

## References

[B1] Abdelfattah A. Malacrinò A. Wisniewski M. Cacciola S. O. Schena L. (2018). Metabarcoding: a powerful tool to investigate microbial communities and shape future plant protection strategies. *Biol. Control* 120 1–10. 10.1016/j.biocontrol.2017.07.009

[B2] Abdelfattah A. Nicosia M. G. L. D. Cacciola S. O. Droby S. Schena L. (2015). Metabarcoding analysis of fungal diversity in the phyllosphere and carposphere of olive (*Olea europaea*). *PLoS One* 10:e0131069. 10.1371/journal.pone.0131069 26132745 PMC4489200

[B3] Abdelfattah A. Wisniewski M. Nicosia M. G. L. D. Cacciola S. O. Schena L. (2016). Metagenomic analysis of fungal diversity on strawberry plants and the effect of management practices on the fungal community structure of aerial organs. *PLoS One* 11:e0160470. 10.1371/journal.pone.0160470a 27490110 PMC4973904

[B4] Aiello D. Restuccia C. Stefani E. Vitale A. Cirvilleri G. (2019). Postharvest biocontrol ability of *Pseudomonas synxantha* against *Monilinia fructicola* and *Monilinia fructigena* on stone fruit. *Postharvest Biol. Technol.* 149 83–89. 10.1016/j.postharvbio.2018.11.020

[B5] Altindag M. Sahin M. Esitken A. Ercisli S. Guleryuz M. Donmez M. F. (2006). Biological control of brown rot (*Moniliana laxa* Ehr.) on apricot (*Prunus armeniaca* L. cv. Hacıhaliloğlu) by *Bacillus*, *Burkholdria*, and *Pseudomonas* application under in vitro and in vivo conditions. *Biol. Control* 38 369–372. 10.1016/j.biocontrol.2006.04.015

[B6] Amborabé B. E. Fleurat-Lessard P. Chollet J. F. Roblin G. (2002). Antifungal effects of salicylic acid and other benzoic acid derivatives towards *Eutypa lata*: structure–activity relationship. *Plant Physiol. Biochem.* 40 1051–1060. 10.1016/S0981-9428(02)01470-5

[B7] Barata A. Malfeito-Ferreira M. Loureiro V. (2012). The microbial ecology of wine grape berries. *Int. J. Food Microbiol.* 153 243–259. 10.1016/j.ijfoodmicro.2011.11.025 22189021

[B8] Bonaterra A. Mari M. Casalini L. Montesinos E. (2003). Biological control of *Monilinia laxa* and *Rhizopus stolonifer* in postharvest of stone fruit by *Pantoea agglomerans* EPS125 and putative mechanisms of antagonism. *Int. J. Food Microbiol.* 84 93–104. 10.1016/S0168-1605(02)00403-8 12781959

[B9] Callahan B. J. McMurdie P. J. Rosen M. J. Han A. W. Johnson A. J. A. Holmes S. P. (2016). DADA2: high-resolution sample inference from Illumina amplicon data. *Nat. Methods* 13:581. 10.1038/nmeth.3869 27214047 PMC4927377

[B10] Cannon P. F. Kirk P. M. (2007). *Fungal Families of the World.* Wallingford: CABI.

[B11] Chin-A-Woeng T. F. Bloemberg G. V. Lugtenberg B. J. (2003). Phenazines and their role in biocontrol by *Pseudomonas* bacteria. *New Phytol.* 157 503–523. 10.1046/j.1469-8137.2003.00686.x 33873412

[B12] da Rocha Neto A. C. Maraschin M. Di Piero R. M. (2015). Antifungal activity of salicylic acid against *Penicillium expansum* and its possible mechanisms of action. *Int. J. Food Microbiol.* 215 64–70. 10.1016/j.ijfoodmicro.2015.08.018 26340673

[B13] D’aes J. De Maeyer K. Pauwelyn E. Höfte M. (2010). Biosurfactants in plant–*Pseudomonas* interactions and their importance to biocontrol. *Environ. Microbiol. Rep.* 2 359–372. 10.1111/j.1758-2229.2009.00104.x 23766108

[B14] de Souza J. T. Raaijmakers J. M. (2003). Polymorphisms within the prnD and pltC genes from pyrrolnitrin and pyoluteorin-producing *Pseudomonas* and *Burkholderia* spp. *FEMS Microbiol. Ecol.* 43 21–34. 10.1111/j.1574-6941.2003.tb01042.x 19719693

[B15] Di Francesco A. Ugolini L. D’Aquino S. Pagnotta E. Mari M. (2017). Biocontrol of *Monilinia laxa* by *Aureobasidium pullulans* strains: insights on competition for nutrients and space. *Int. J. Food Microbiol.* 248 32–38. 10.1016/j.ijfoodmicro.2017.02.007 28242420

[B16] Dimkić I. Berić T. Stević T. Pavlović S. Šavikin K. Fira D. (2015). Additive and synergistic effects of *Bacillus* spp. isolates and essential oils on the control of phytopathogenic and saprophytic fungi from medicinal plants and marigold seeds. *Biol. Control* 87 6–13. 10.1016/j.biocontrol.2015.04.011

[B17] Dimkić I. Živković S. Berić T. Ivanović Ž Gavrilović V. Stanković S. (2013). Characterization and evaluation of two *Bacillus* strains, SS-12.6 and SS-13.1, as potential agents for the control of phytopathogenic bacteria and fungi. *Biol. Control* 65 312–321. 10.1016/j.biocontrol.2013.03.012

[B18] Dionísio A. P. Molina G. de Carvalho D. S. Dos Santos R. Bicas J. L. Pastore G. M. (2012). “Natural flavourings from biotechnology for foods and beverages,”,” in *Natural Food Additives, Ingredients and Flavourings*, eds Baines D. Seal R. (Cambridge: Woodhead Publishing), 231–259. 10.1533/9780857095725.1.231

[B19] El-Mohamedy R. S. Abdel-Kader M. M. Abd-El-Kareem F. El-Mougy N. S. (2013). Inhibitory effect of antagonistic bio-agents and chitosan on the growth of tomato root rot pathogens *in vitro*. *Int. J. Agric. Technol.* 9 1521–1533.

[B20] El-Sayed W. El-Megeed M. A. El-Razik A. A. Soliman K. H. Ibrahim S. A. (2008). Isolation and identification of phenazine-1-carboxylic acid from different *Pesudomonas* isolates and its biological activity against *Alternaria solani*. *Res. J. Agric. Biol. Sci.* 4 892–901.

[B21] FAOSTAT (2017). *Food and Agriculture Organization of the United Nations.* Rome: FAOSTAT.

[B22] Fogarty R. V. Tobin J. M. (1996). Fungal melanins and their interactions with metals. *Enzyme Microb. Technol.* 19 311–317. 10.1016/0141-0229(96)00002-6 8987489

[B23] Frases S. Salazar A. Dadachova E. Casadevall A. (2007). *Cryptococcus neoformans* can utilize the bacterial melanin precursor homogentistic acid for fungal melanogenesis. *Appl. Environ. Microbiol.* 73 615–621. 10.1128/AEM.01947-06 17098915 PMC1796974

[B24] Garrido-Sanz D. Arrebola E. Martínez-Granero F. García-Méndez S. Muriel C. Blanco-Romero E. (2017). Classification of isolates from the *Pseudomonas fluorescens* complex into phylogenomic groups based in group-specific markers. *Front. Microbiol.* 8:413. 10.3389/fmicb.2017.00413 28360897 PMC5350142

[B25] Giorgio A. De Stradis A. Lo Cantore P. Iacobellis N. S. (2015). Biocide effects of volatile organic compounds produced by potential biocontrol rhizobacteria on *Sclerotinia sclerotiorum*. *Front. Microbiol.* 6:1056. 10.3389/fmicb.2015.01056 26500617 PMC4594563

[B26] Graça A. Santo D. Esteves E. Nunes C. Abadias M. Quintas C. (2015). Evaluation of microbial quality and yeast diversity in fresh-cut apple. *Food Microbiol.* 51 179–185. 10.1016/j.fm.2015.06.003 26187843

[B27] Gross H. Loper J. E. (2009). Genomics of secondary metabolite production by *Pseudomonas* spp. *Nat. Prod. Rep.* 26 1408–1446. 10.1039/b817075b 19844639

[B28] Gschaedler A. (2017). Contribution of non-conventional yeasts in alcoholic beverages. *Curr. Opin. Food Sci.* 13 73–77. 10.1016/j.cofs.2017.02.004

[B29] Haas J. C. Street N. R. Sjödin A. Lee N. M. Högberg M. N. Näsholm T. (2018). Microbial community response to growing season and plant nutrient optimisation in a boreal Norway spruce forest. *Soil Biol. Biochem.* 125 197–209. 10.1016/j.soilbio.2018.07.005

[B30] Henson J. M. Butler M. J. Day A. W. (1999). The dark side of the mycelium: melanins of phytopathogenic fungi. *Annu. Rev. Phytopathol.* 37 447–471. 10.1146/annurev.phyto.37.1.447 11701831

[B31] Hernández-León R. Rojas-Solís D. Contreras-Pérez M. del Carmen Orozco-Mosqueda M. Macías-Rodríguez I. Reyes-de la Cruz H. (2015). Characterization of the antifungal and plant growth-promoting effects of diffusible and volatile organic compounds produced by *Pseudomonas fluorescens* strains. *Biol. Control* 81 83–92. 10.1016/j.biocontrol.2014.11.011

[B32] Hrustić J. Delibašić G. Stanković I. Grahovac M. Krstić B. Bulajić A. (2015). *Monilinia* causing brown rot of stone fruit in Serbia. *Plant Dis.* 99 709–717. 10.1094/PDIS-07-14-0732-RE 30699676

[B33] Jain R. Pandey A. (2016). A phenazine-1-carboxylic acid producing polyextremophilic *Pseudomonas chlororaphis* (MCC2693) strain, isolated from mountain ecosystem, possesses biocontrol and plant growth promotion abilities. *Microbiol. Res.* 190 63–71. 10.1016/j.micres.2016.04.017 27394000

[B34] Janisiewicz W. J. Jurick W. M. II Vico I. Peter K. A. Buyer J. S. (2013). Culturable bacteria from plum fruit surfaces and their potential for controlling brown rot after harvest. *Postharvest Biol. Technol.* 76 145–151. 10.1016/j.postharvbio.2012.10.004

[B35] Janisiewicz W. J. Jurick W. M. Peter K. A. Kurtzman C. P. Buyer J. S. (2014). Yeasts associated with plums and their potential for controlling brown rot after harvest. *Yeast* 31 207–218. 10.1002/yea.3009 24687564

[B36] Janisiewicz W. J. Kurtzman C. P. Buyer J. S. (2010). Yeasts associated with nectarines and their potential for biological control of brown rot. *Yeast* 27 389–398. 10.1002/yea.1763 20225339

[B37] Janisiewicz W. J. Roitman J. (1988). Biological control of blue mold and gray mold on apple and pear with *Pseudomonas cepacia*. *Phytopathology* 78 1697–1700.

[B38] Janisiewicz W. J. Tworkoski T. J. Kurtzman C. P. (2001). Biocontrol potential of *Metchnikowia pulcherrima* strains against blue mold of apple. *Phytopathology* 91 1098–1108. 10.1094/PHYTO.2001.91.11.1098 18943447

[B39] Janisiewicz W. J. Tworkoski T. J. Sharer C. (2000). Characterizing the mechanism of biological control of postharvest diseases on fruits with a simple method to study competition for nutrients. *Phytopathology* 90 1196–1200. 10.1094/PHYTO.2000.90.11.1196 18944420

[B40] Leff B. Ramankutty N. Foley J. A. (2004). Geographic distribution of major crops across the world. *Glob. Biogeochem. Cycles* 18:GB1009 10.1029/2003GB002108

[B41] Lino O. L. Pacheco I. Mercier V. Faoro F. Bassi D. Bornard I. (2016). Brown rot strikes *Prunus* fruit: an ancient fight almost always lost. *J. Agric. Food Chem.* 64 4029–4047. 10.1021/acs.jafc.6b00104 27133976

[B42] Machado A. P. Vivi V. K. Tavares J. S. Gueiros Filho F. J. Fischman O. (2010b). Antibiosis and dark-pigments secretion by the phytopathogenic and environmental fungal species after interaction in vitro with a *Bacillus subtilis* isolate. *Braz. Arch. Biol. Technol.* 53 997–1004. 10.1590/S1516-89132010000500001

[B43] Machado A. P. Anzai M. Fischman O. (2010a). Bacillus subtilis induces morphological changes in *Fonsecaea pedrosoi* in vitro resulting in more resistant fungal forms *in vivo*. *J. Venom. Anim. Toxins Incl. Trop. Dis.* 16 592–598. 10.1590/S1678-91992010000400009

[B44] Magan N. (2006). “Ecophysiology of biocontrol agents for improved competence in the phyllosphere,” in *Microbial Ecology of Aerial Plant Surfaces*, eds Bailey M. J. Lilley A. K. Timms-Wilson T. M. Spencer Phillips P. T. N. (Wallingford: CAB International), 149–164. 10.1079/9781845930615.0149

[B45] Manso T. Nunes C. (2011). *Metschnikowia andauensis* as a new biocontrol agent of fruit postharvest diseases. *Postharvest Biol. Technol.* 61 64–71. 10.1016/j.postharvbio.2011.02.004

[B46] Marengo E. Gennaro M. C. Gianotti V. (2001). A simplex-optimized chromatographic separation of fourteen cosmetic preservatives: analysis of commercial products. *J. Chromatogr. Sci.* 39 339–344. 10.1093/chromsci/39.8.339 11513276

[B47] Mari M. Martini C. Guidarelli M. Neri F. (2012). Postharvest biocontrol of *Monilinia laxa*, *Monilinia fructicola* and *Monilinia fructigena* on stone fruit by two *Aureobasidium pullulans* strains. *Biolog. Control.* 60 132–140. 10.1016/j.biocontrol.2011.10.013

[B48] Materatski P. Varanda C. Carvalho T. Dias A. B. Campos M. D. Rei F. (2019). Spatial and temporal variation of fungal endophytic richness and diversity associated to the phyllosphere of olive cultivars. *Fungal Biol.* 123 66–76. 10.1016/j.funbio.2018.11.004 30654959

[B49] Matković M. (2015). Possibilities of plum cultivation in the republic of Serbia. *Econ. Agric.* 62 1045–1060. 10.5937/ekopolj1504045m

[B50] Mukherjee K. Mandal S. Mukhopadhyay B. Mandal N. C. Sil A. K. (2014). Bioactive compound from *Pseudomonas synxantha* inhibits the growth of *Mycobacteria*. *Microbiol. Res.* 169 794–802. 10.1016/j.micres.2013.12.005 24439826

[B51] Müller T. Behrendt U. Ruppel S. von, der Waydbrink G. Müller M. E. (2016). Fluorescent pseudomonads in the phyllosphere of wheat: potential antagonists against fungal phytopathogens. *Curr. Microbiol.* 72 383–389. 10.1007/s00284-015-0966-8 26687461

[B52] Müller T. Ruppel S. (2014). Progress in cultivation-independent phyllosphere microbiology. *FEMS Microbiol. Ecol.* 87 2–17. 10.1111/1574-6941.12198 24003903 PMC3906827

[B53] Newton A. C. Gravouil C. Fountaine J. M. (2010). Managing the ecology of foliar pathogens: ecological tolerance in crops. *Ann. Appl. Biol.* 157 343–359. 10.1111/j.1744-7348.2010.00437.x

[B54] Nosanchuk J. D. Casadevall A. (2003). The contribution of melanin to microbial pathogenesis. *Cell Microbiol.* 5 203–223. 10.1046/j.1462-5814.2003.00268.x 12675679

[B55] Ottesen A. R. Peña A. G. White J. R. Pettengill J. B. Li C. Allard S. (2013). Baseline survey of the anatomical microbial ecology of an important food plant: *Solanum lycopersicum* (tomato). *BMC Microbiol.* 13:114. 10.1186/1471-2180-13-114 23705801 PMC3680157

[B56] Panahirad S. Zaare-Nahandi F. Safaralizadeh R. Alizadeh-Salteh S. (2013). Postharvest control of *Rhizopus stolonifer* in peach (*Prunus persica* L. *B atsch*) fruits using salicylic acid. *J. Food Saf.* 32 502–507. 10.1111/jfs.12013 29044849

[B57] Parejko J. A. Mavrodi D. V. Mavrodi O. V. Weller D. M. Thomashow L. S. (2012). Population structure and diversity of phenazine-1-carboxylic acid producing fluorescent *Pseudomonas* spp. from dryland cereal fields of central Washington State (USA). *Microb. Ecol.* 64 226–241. 10.1007/s00248-012-0015-0 22383119

[B58] Penuelas J. Rico L. Ogaya R. Jump A. S. Terradas J. (2012). Summer season and longterm drought increase the richness of bacteria and fungi in the foliar phyllosphere of *Quercus ilex* in a mixed Mediterranean forest. *Plant Biol.* 14 565–575. 10.1111/j.1438-8677.2011.00532.x 22289059

[B59] Petróczy M. Szigethy A. Palkovics L. (2012). *Monilinia* species in Hungary: morphology, culture characteristics, and molecular analysis. *Trees* 26 153–164. 10.1007/s00468-011-0622-2

[B60] Pinto C. Pinho D. Sousa S. Pinheiro M. Egas C. Gomes A. C. (2014). Unravelling the diversity of grapevine microbiome. *PLoS One* 9:e85622. 10.1371/journal.pone.0085622 24454903 PMC3894198

[B61] Poniatowska A. Michalecka M. Bielenin A. (2013). Characteristic of *Monilinia* spp. fungi causing brown rot of pome and stone fruits in Poland. *Eur. J. Plant. Pathol.* 135 855–865. 10.1007/s10658-012-0130-2

[B62] Quast C. Pruesse E. Yilmaz P. Gerken J. Schweer T. Yarza P. (2013). The SILVA ribosomal RNA gene database project: improved data processing and web-based tools. *Nucleic Acids Res.* 41 D590–D596. 10.1093/nar/gks1219 23193283 PMC3531112

[B63] R Development Core Team (2012). *R: A Language and Environment for Statistical Computing.* Vienna: R foundation for Statistical Computing.

[B64] Raaijmakers J. M. Weller D. M. Thomashow L. S. (1997). Frequency of antibiotic-producing *Pseudomonas* spp. in natural environments. *Appl. Environ. Microbiol.* 63 881–887. 16535555 10.1128/aem.63.3.881-887.1997 PMC1389120

[B65] Raio A. Reveglia P. Puopolo G. Cimmino A. Danti R. Evidente A. (2017). Involvement of phenazine-1-carboxylic acid in the interaction between *Pseudomonas chlororaphis* subsp. *aureofaciens* strain M71 and *Seiridium cardinale* in vivo. *Microbiol. Res.* 199 49–56. 10.1016/j.micres.2017.03.003 28454709

[B66] Ramette A. Frapolli M. Défago G. Moënne-Loccoz Y. (2003). Phylogeny of HCN synthase-encoding hcnBC genes in biocontrol fluorescent pseudomonads and its relationship with host plant species and HCN synthesis ability. *Mol. Plant Microb. Interact.* 16 525–535. 10.1094/MPMI.2003.16.6.525 12795378

[B67] RHMZ (2017). *Meteorological Yearbook 1.* Belgrade: Climatological data.

[B68] Rungjindamai N. Jeffries P. Xu X. M. (2014). Epidemiology and management of brown rot on stone fruit caused by *Monilinia laxa*. *Eur. J. Plant Pathol.* 140 1–17. 10.1007/s10658-014-0452-3

[B69] Rungjindamai N. Xu X. M. Jeffries P. (2013). Identification and characterization of new microbial antagonists for biocontrol of *Monilinia laxa*, the causal agent of brown rot on stone fruit. *Agronomy* 3 685–703. 10.3390/agronomy3040685

[B70] Santo D. E. Galego L. Gonçalves T. Quintas C. (2012). Yeast diversity in the Mediterranean strawberry tree (*Arbutus unedo* L.) fruits’ fermentations. *Food Res. Int.* 47 45–50. 10.1016/j.foodres.2012.01.009

[B71] Santoyo G. Orozco-Mosqueda M. D. C. Govindappa M. (2012). Mechanisms of biocontrol and plant growth-promoting activity in soil bacterial species of *Bacillus* and *Pseudomonas*: a review. *Biocontrol Sci. Technol.* 22 855–872. 10.1080/09583157.2012.694413 18845426

[B72] Sarkar S. F. Guttman D. S. (2004). Evolution of the core genome of *Pseudomonas syringae*, a highly clonal, endemic plant pathogen. *Appl. Environ. Microbiol.* 70 1999–2012. 10.1128/AEM.70.4.1999-2012.2004 15066790 PMC383139

[B73] Schmieder R. Edwards R. (2011). Quality control and preprocessing of metagenomic datasets. *Bioinformatics* 27 863–864. 10.1093/bioinformatics/btr026 21278185 PMC3051327

[B74] Shivas R. G. Grice K. R. E. Young A. J. (2011). Ramichloridium spp. on Musa in northern queensland: introducing *Ramichloridium ducassei* sp. nov. on leaf streaks of *Ducasse banana*. *Australas. Plant Path.* 40 61–65. 10.1007/s13313-010-0014-x

[B75] Sylla J. Alsanius B. W. Krüger E. Reineke A. Strohmeier S. Wohanka W. (2013). Leaf microbiota of strawberries as affected by biological control agents. *Phytopathology* 103 1001–1011. 10.1094/PHYTO-01-13-0014-R 24020904

[B76] Tedersoo L. Anslan S. Bahram M. Põlme S. Riit T. Liiv I. (2015). Shotgun metagenomes and multiple primer pair-barcode combinations of amplicons reveal biases in metabarcoding analyses of fungi. *MycoKeys* 10 1–43. 10.3897/mycokeys.10.4852

[B77] Thompson J. D. Gibson T. J. Plewniak F. Jeanmougin F. Higgins D. G. (1997). The CLUSTAL_X windows interface: flexible strategies for multiple sequence alignment aided by quality analysis tools. *Nucleic Acids Res.* 25 4876–4882. 10.1093/nar/25.24.4876 9396791 PMC147148

[B78] Unković N. Erić S. Šarić K. Stupar M. Savković Ž Stanković S. (2017). Biogenesis of secondary mycogenic minerals related to wall paintings deterioration process. *Micron* 100 1–9. 10.1016/j.micron.2017.04.004 28448830

[B79] Vasić V. Gašić U. Stanković D. Lušić D. Vukić-Lušić D. Milojković-Opsenica D. (2019). Towards better quality criteria of European honeydew honey: phenolic profile and antioxidant capacity. *Food Chem.* 274 629–641. 10.1016/j.foodchem.2018.09.045 30372988

[B80] Wechter W. P. Begum D. Presting G. Kim J. J. Wing R. A. Kluepfel D. A. (2002). Physical mapping, BAC-end sequence analysis, and marker tagging of the soilborne nematicidal bacterium, *Pseudomonas synxantha* BG33R. *Omics* 6 11–21. 10.1089/15362310252780807 11881828

[B81] Weller D. M. (2007). *Pseudomonas* biocontrol agents of soilborne pathogens: looking back over 30 years. *Phytopathology* 97 250–256. 10.1094/PHYTO-97-2-0250 18944383

[B82] White T. J. Bruns T. Lee S. J. W. T. Taylor J. L. (1990). “Amplification and direct sequencing of fungal ribosomal RNA genes for phylogenetics,” in *PCR Protocols - a Guide to Methods and Applications*, eds Innis M. A. Gelfand D. H. Sninsky J. J. White T. J. (London: Academic Press), 482.

