# Supplementary material for: Phyllosphere Fungal Communities of Plum and Antifungal Activity of Indigenous Phenazine-Producing Pseudomonas synxantha Against Monilinia laxa
Source: Front Microbiol. 2019 Oct 1;10:2287. doi: 10.3389/fmicb.2019.02287 (PMC6779809; doi:10.3389/fmicb.2019.02287)
Supplement: Supplementary file 1 [file Table_1.DOCX]

Supplementary Material

**Supplementary Table S1.** Relative abundances (RA) of the fungal taxa in all samples at the phylum (sheet 1), family (sheet 2), genus (sheet 3) and species (sheet 4) levels.

**Supplementary Table S2.** The total number of bases, reads, GC (%), Q20 (%), and Q30 (%) scores

| **Sample ID** | **Total read bases (bp)** | **Total reads** | **GC (%)** | **AT (%)** | **Q20 (%)** | **Q30 (%)** |
| --- | --- | --- | --- | --- | --- | --- |
| **May** | | | | | | |
| 9-Lepotica-1 | 90,948,956 | 302,156 | 50.63 | 49.37 | 83.36 | 74.62 |
| 10-Rodna-1 | 80,082,254 | 266,054 | 50.34 | 49.66 | 82.41 | 73.81 |
| 11-Požegača-1 | 82,458,348 | 273,948 | 49.47 | 50.53 | 82.71 | 74.38 |
| 12-Ranka-1 | 90,960,394 | 302,194 | 48.92 | 51.08 | 84.02 | 75.49 |
| **July** | | | | | | |
| 13-Lepotica-2 | 89,856,326 | 298,526 | 50.01 | 49.99 | 83.27 | 74.73 |
| 14-Rodna-2 | 75,508,860 | 250,860 | 54.95 | 45.05 | 79.34 | 70.45 |
| 15-Požegača-2 | 74,439,106 | 247,306 | 49.47 | 50.53 | 81.53 | 73.11 |
| 16-Ranka-2 | 81,299,498 | 270,098 | 50.15 | 49.85 | 82.20 | 73.84 |

Total reads: Total number of reads. In Illumina paired-end sequencing, read1 and read2 are added. Q20(%) and Q30(%) is a ratio of reads that have phred quality score of over 20 and 30, respectively.

**Figure legends:**

**Supplementary Figure S1.** Venn diagrams illustrating OTUs distribution among plum cultivars in both phenological stages May (A) and July (B).

**Supplementary Figure S2.** Relative abundance (RA) of the fungal taxa associated with four plum cultivars at two phenological stages (May and July) on phylum (A) and family (B) levels. Only taxa with total percentage of RA values above 2% were presented.

**Supplementary** **Figure S3.** Phylogenetic relationships of *Monilinia* *laxa* isolates based on amplified ribosomal DNA internal transcribed spacer regions; ITS 1 including partial 18S+ internal transcribed spacer 1 and partial 5.8S (A) and ITS 2 including partial 5.8S + internal transcribed spacer 2+ partial 28S (B). The phylogenetic trees were constructed by the neighbor-joining method, which are rooted by *Sclerotinia sclerotiorum* as outgroup and the distances were calculated with the Kimura two-parameter model. Bootstrap values are given for each node, with 1000 replicates.

**Supplementary** **Figure S4.** Neighbor-joining phylogenetic tree based on the concatenated sequences of the genes 16S rRNA and *gyrB*. The concatenated sequence of 16S rRNA and *gyrB* of *P. aeruginosa* ATCC 15692 was used as an outgroup.

**Fig. S1**


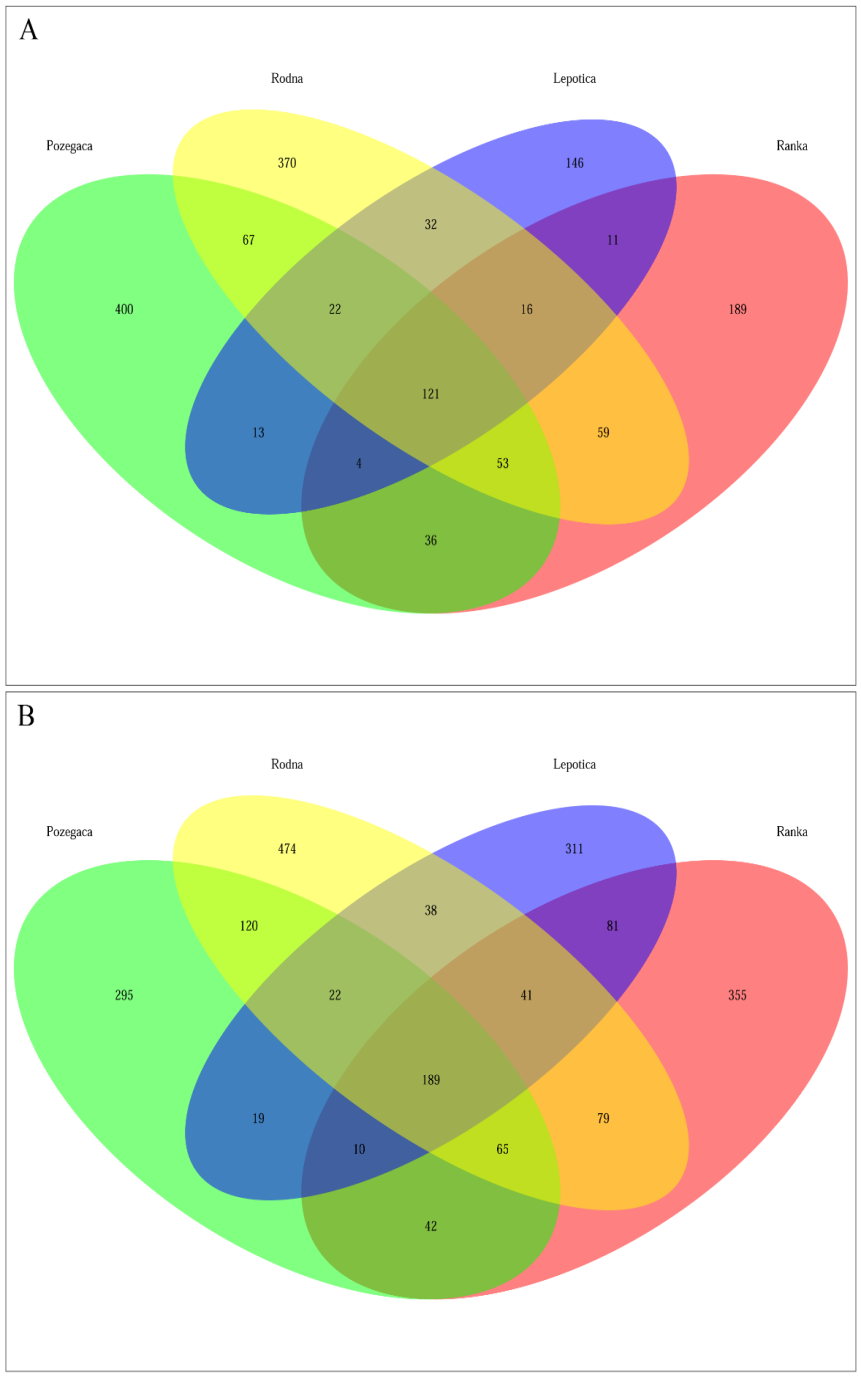


**Fig. S2**


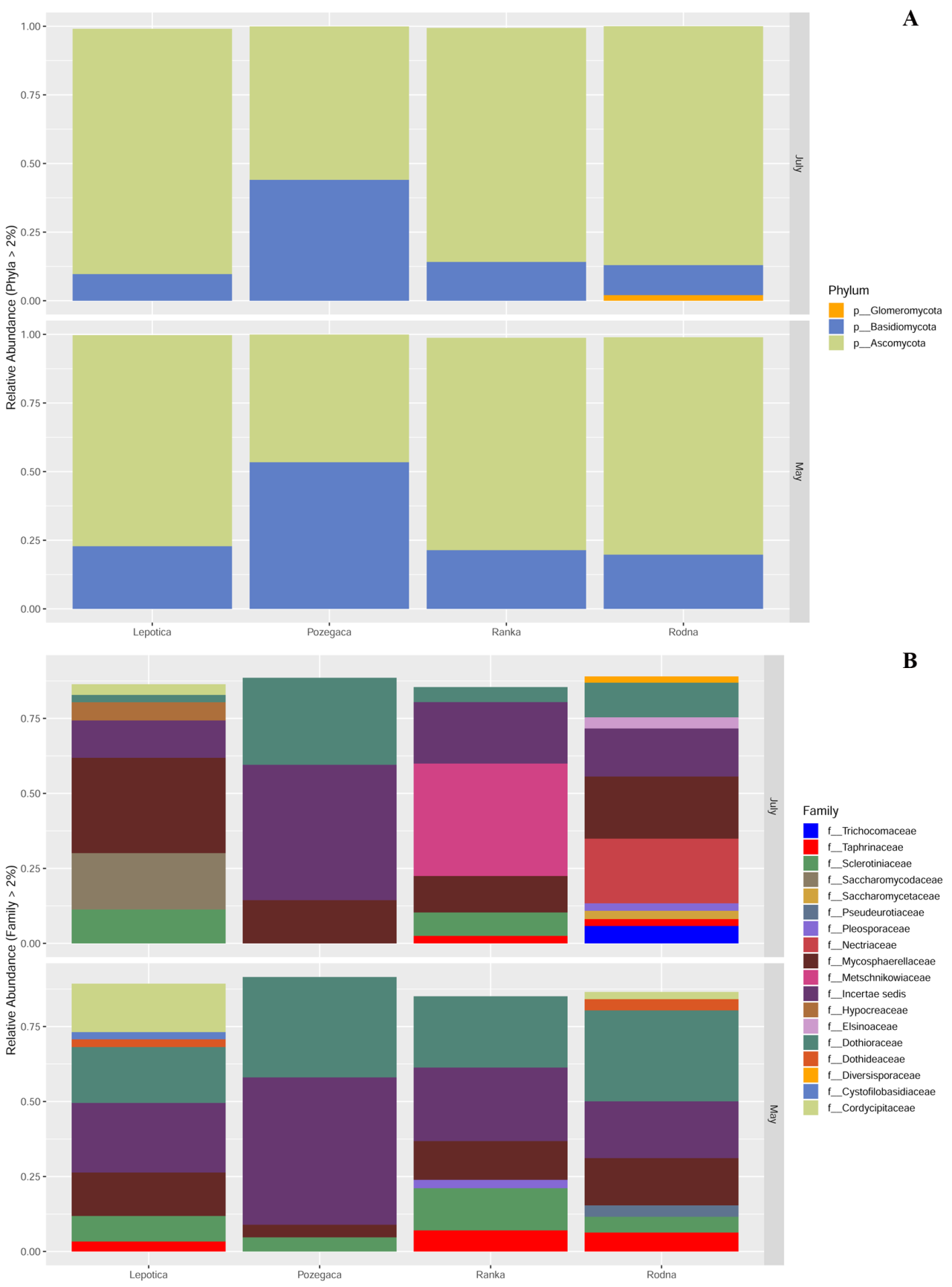


**Fig. S3**

**
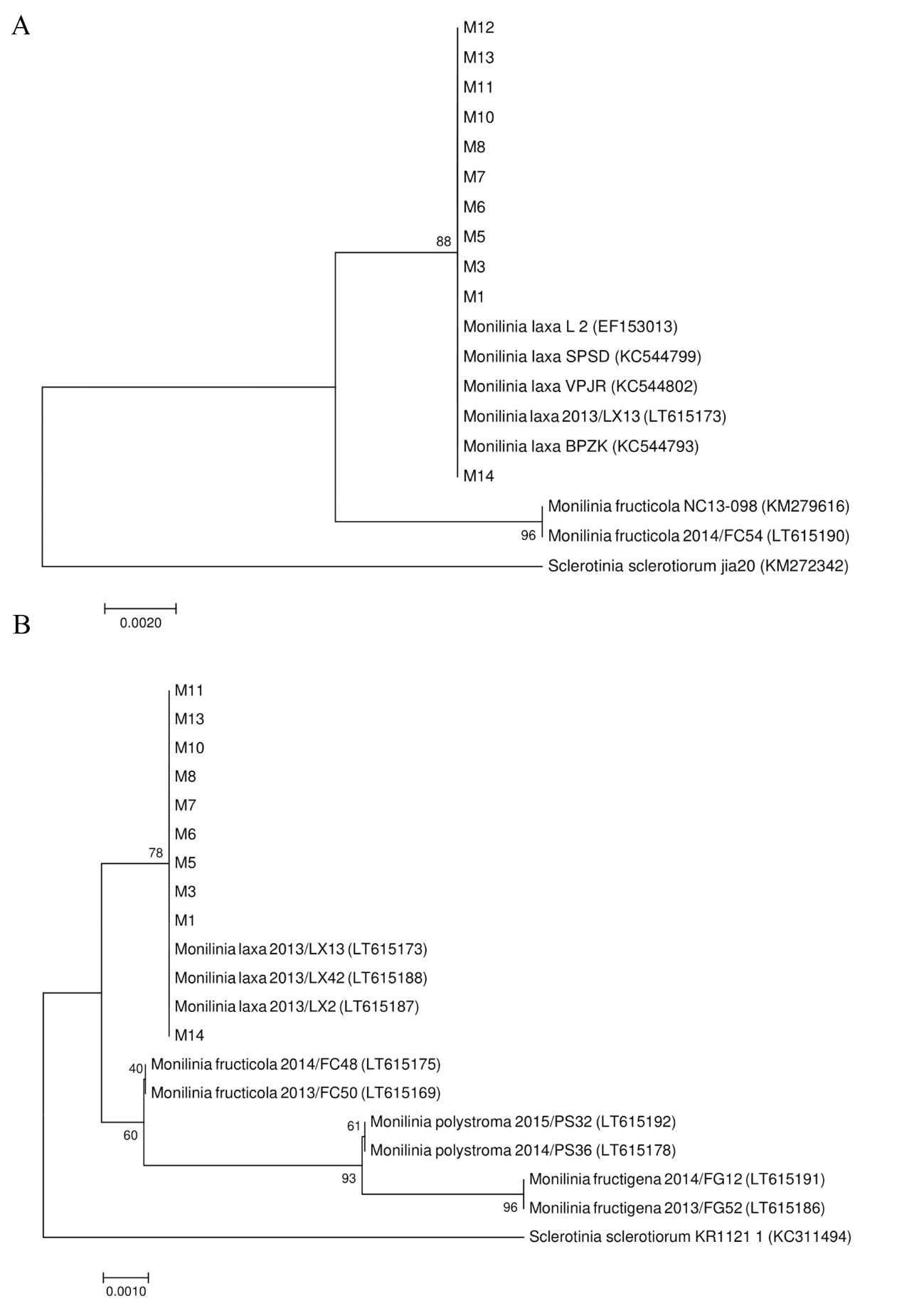
**

**Fig S4.**

**

**
